# Design, Synthesis, and Bioactivity Assessment of Modified Vemurafenib Analog

**DOI:** 10.3390/ph18081161

**Published:** 2025-08-05

**Authors:** Fabiana Sélos Guerra, Rosana Helena Coimbra Nogueira de Freitas, Florina Moldovan, David Rodrigues da Rocha, Renato Sampaio Carvalho, Patricia Dias Fernandes

**Affiliations:** 1 Laboratório de Farmacologia da Dor e da Inflamação, Instituto de Ciências Biomédicas, Universidade Federal do Rio de Janeiro, Rio de Janeiro 21941-902, RJ, Brazil; fabianasellos@hotmail.com (F.S.G.); renato.carvalho@icb.ufrj.br (R.S.C.); 2 Laboratório de Síntese de Substâncias de Interesse Biológico (SiMIB), Instituto de Quıímica, Campus do Valonguinho, Universidade Federal Fluminense, Niterói 24020-141, RJ, Brazil; freitasrh@yahoo.com.br (R.H.C.N.d.F.); davidrrocha@vm.uff.br (D.R.d.R.); 3 Instituto de Química, Universidade do Estado do Rio de Janeiro, Rio de Janeiro 20550-900, RJ, Brazil; 4 Centre Hospitalier Universitaire St. Justine, Université de Montréal, Montréal, QC H3T 1C5, Canada; florina.moldovan@umontreal.ca

**Keywords:** melanoma, cancer, azaindole, *N*-acylhydrazone

## Abstract

**Background:** Metastatic melanoma is a highly aggressive malignancy with poor prognoses and frequent resistance to conventional chemotherapy. Approximately 40% of melanoma cases carry the BRAF^V600E^ mutation, for which vemurafenib, a selective BRAF^V600E^ inhibitor, is approved. Despite initial clinical benefits, vemurafenib often leads to drug resistance and relapse, highlighting the need for improved therapeutic strategies. **Objectives, methods:** In this study, we designed, synthesized, and characterized five novel vemurafenib analogs—RF-86A, RF-87A, RF-94A, RF-94B, and RF-96B—with the aim of enhancing anti-proliferative and anti-metastatic effects against human melanoma cells. **Results:** All compounds induced apoptosis in BRAF^V600E^-mutated A375 cells, with RF-86A displaying the lowest IC_50_ value among the series, comparable to that of vemurafenib. Moreover, RF-86A exhibited the highest selectivity index, as determined using HEK293T cells as a non-tumorigenic control. Additionally, migration assays and gelatin zymography demonstrated that the analogs, unlike vemurafenib, significantly inhibited matrix metalloproteinases MMP-2 and MMP-9, key enzymes involved in tumor invasion and metastasis. **Conclusions:** These findings suggest that structural modifications to the vemurafenib scaffold may improve therapeutic efficacy and offer a promising strategy to overcome acquired resistance.

## 1. Introduction

Patients with metastatic melanoma face a poor prognosis due to the limited effectiveness of chemotherapy. Metastasis—the leading cause of cancer-related deaths—is a complex, multistep process that involves cell adhesion, motility, extracellular matrix (ECM) degradation, angiogenesis, and tissue invasion [1].

Approximately 50% of melanomas carry activating mutations in the *BRAF* gene, with over 90% of these being the V600E variant. This mutation produces a constitutively active BRAF protein that drives continuous activation of the mitogen-activated protein kinase (MAPK) signaling pathway. Activation of this pathway plays a key role in promoting melanoma cell proliferation, inhibiting apoptosis, and driving tumor progression [2].

Vemurafenib is an oral BRAF inhibitor approved for the treatment of patients with inoperable or metastatic melanoma harboring the *BRAF* V600E mutation. It selectively binds to the ATP-binding site of the mutant BRAF V600E kinase. By doing so, it inhibits its kinase activity, effectively blocking downstream signaling through MEK and ERK, leading to dose-dependent anti-proliferative and pro-apoptotic effects on melanoma cells [3,4]. While vemurafenib offers significant clinical benefits, its effectiveness is often limited by the rapid development of resistance, leading to disease relapse. Additionally, treatment is associated with notable side effects, including fatigue, joint pain, headaches, skin toxicity, and the emergence of secondary skin tumors [4].

To overcome these challenges, the discovery of new BRAF^V600E^ inhibitors with improved efficacy and reduced toxicity is essential. Using vemurafenib as a starting point, our research group synthesized five novel analogs—**2a** (RF-86A), **2d** (RF-87A), **2c** (RF-94A), **2b** (RF-94B), and **2e** (RF-96B)—with structural modifications designed to enhance anti-proliferative and anti-metastatic effects in human melanoma cells.

**Figure 1:**
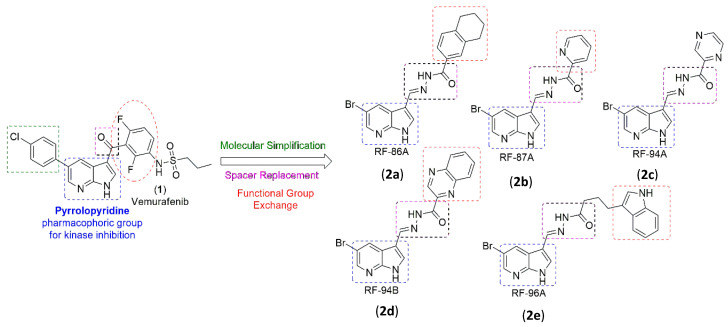
Design concept of new pyrrolopyridine-*N*-acylhydrazone derivatives (**2a**–**e**).

The design concept of the molecules in this work is based on the principle of Molecular Simplification [5], using the BRAF inhibitor vemurafenib (**1**, Figure 1) as a reference. The pyrrolopyridine or azaindole core—recognized in the literature as a key pharmacophore for kinase inhibition [6,7]—was retained in all compounds. The amide group present in the parent compound was replaced by an *N*-acylhydrazone moiety, a well-established privileged structure [8] with documented bioactivity [9], leading to the first reported series of azaindole–*N*-acylhydrazone derivatives. The 2,6-difluorophenyl group was substituted with either a tetrahydronaphthalene or a nitrogen-containing heterocycle. The tetrahydronaphthalene moiety (RF-86A or **2a**) was selected for its ability to occupy hydrophobic regions within the ATP-binding pocket, while maintaining a lipophilic profile similar to the original aryl ring. In compounds **2b–2e** (Figure 1), the aryl ring was replaced by nitrogenous heterocycles. These fragments were chosen specifically to increase the potential for hydrogen bonding interactions with the kinase active site, thereby aiming to improve both binding affinity and selectivity. Additionally, their polar nature contributes to enhancing the aqueous solubility of the compounds. This series also allowed us to explore whether the number and position of nitrogen atoms in the heterocycle correlate with differences in cytotoxic activity.

In this study, we describe the rational design, chemical synthesis, and in vitro pharmacological evaluation of these novel vemurafenib analogs. We present evidence of their cytotoxic, anti-invasive, and anti-metastatic activities against human melanoma cells.

## 2. Results

### 2.1. Analogs Synthesis

**Scheme 1:**
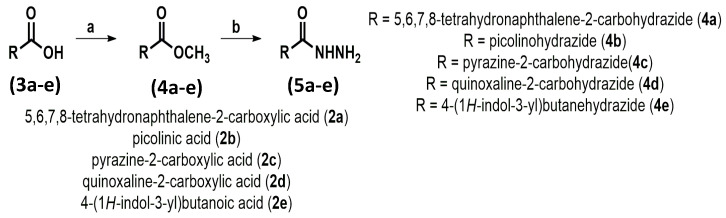
Synthesis of hydrazides (**4a**–**e**). Reagents and Conditions (a) MeOH, MePh, H_2_SO_4_ cat., 100 °C, reflux, 24 h, 42–82%; (b) NH_2_NH_2_ 1M in EtOH, 80 °C, reflux, 48 h, 50–63%.

**Scheme 2:**
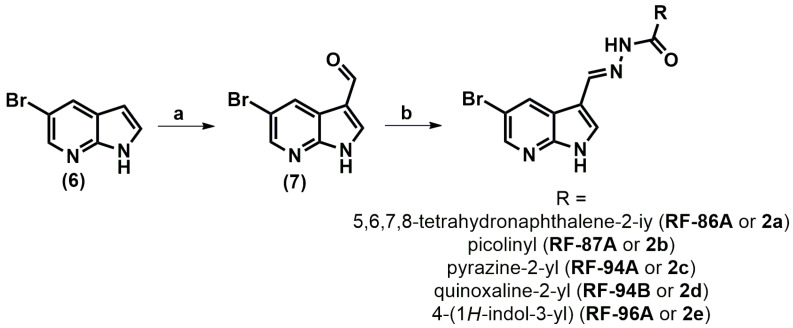
Synthesis of final products (**2a**–**e**). Reagents and Conditions: (a) HMTA, AcOH, and H_2_O (2:1), reflux, 16 h, 66%; (b) hydrazides (**5a**–**e**), EtOH, HClcat., t.a, 6 h, 70–91%.

The synthetic methodology for obtaining the molecules in this work is a convergent synthesis of 5 steps and is presented in Scheme 1 and Scheme 2. Initially, the carboxylic acids (**3a**–**e**) were converted to their respective methyl esters (**4a**–**e**) through the Fischer esterification reaction in methanol, toluene, in acid catalysis promoted by sulfuric acid and refluxing at 100 °C with varying yields [10]. Then, the methyl esters (**4a**–**e**) were added to a 1M ethanolic solution of hydrazine (NH_2_NH_2_) at reflux for 48 h to obtain the corresponding hydrazides (**5a**–**e**) in yields of 50 to 63% (Scheme 2) [11,12].

In parallel, the commercial reagent 5-bromo-7-azaindole (**6**) was regioselectively formylated at position 3 through the Duff reaction by adding hexamethylenetetramine (HMTA) in acetic acid (HOAc) and water at reflux for 16 h to obtain 5-bromo-3-carboxyaldehyde-7-azaindole (**7**) in 66% yield [13]. Finally, the synthesized aldehyde (**7**) was added, separately, to the different hydrazides (**5a**–**e**) obtained in Scheme 1 and in the presence of ethanol and hydrochloric acid catalysis at room temperature for 6 h to form the respective *N*-acylhydrazones (**2a**–**e**) in good yields [11].

### 2.2. Effects on Cell Viability

To assess the effects of the novel vemurafenib analogs on human melanoma A375 cells, a concentration range of 0.01 to 10 μM was evaluated using the MTS cell viability assay. After 24 h of treatment, all analogs significantly reduced A375 cell viability. Notably, only the RF-86A compound displayed an IC_50_ value comparable to that of vemurafenib. Furthermore, RF-86A exhibited the highest selectivity index among the series, as determined using the non-tumor HEK293T cell line (Table 1).

### 2.3. Vemurafenib and Analogs Caused DNA Fragmentation in A375 Cells

**Figure 2:**
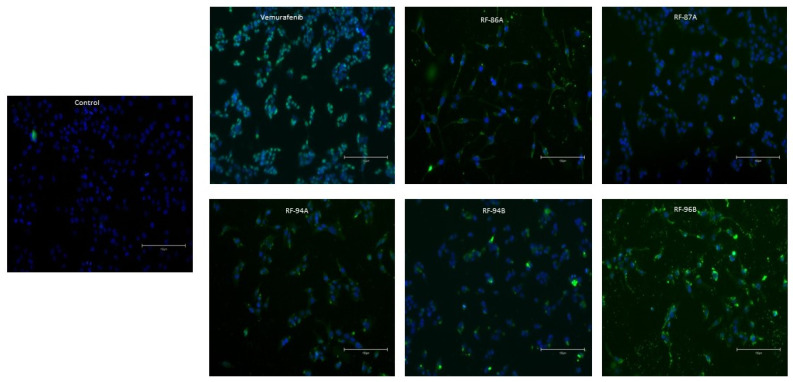
Vemurafenib analogs (**2a**–**e**) induce apoptosis in A375 cells. Apoptosis was detected by TUNEL assay. A double-staining technique was used. TUNEL staining by using an in situ cell death detection kit (fluorescein) for apoptotic cell nuclei and DAPI (blue) staining for all cell nuclei. A375 cells were treated for 24 h with or without (control) 5 μM of the test compounds. TUNEL-positive cells were visualized as indicated by green fluorescence staining. The images were obtained by EVOS M5000 microscope (Thermo Fisher, Waltham, MA, USA). Representative images from three independent experiments are shown. Magnificance: 20×. Scale bar: 150 μm.

To investigate the mechanisms underlying cell death, we performed the TUNEL assay combined with propidium iodide (PI) double staining. The TUNEL assay is a widely used method for detecting DNA fragmentation, a hallmark of apoptosis. As shown in Figure 2, untreated (control) cells displayed blue nuclei, while cells treated with the tested compounds showed green fluorescence, indicating TUNEL-positive apoptotic cells. All compounds induced significant DNA fragmentation after 24 h of treatment at 5 μM. Among them, the analogs RF-94A (**2c**) and RF-96B (**2e**) induced a higher number of apoptotic cells compared to the other analogs, as well as to vemurafenib under the same conditions.

### 2.4. Cell Apoptosis Detected by Cell Morphology Analysis

**Figure 3:**
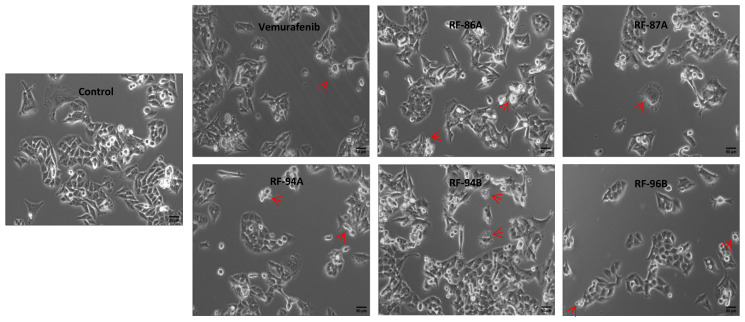
Morphological appearance of A375 cells treated with vemurafenib and analogs (**2a**–**e**) (5 μM) for 24 h. The red arrows indicate apoptotic bodies, showing membrane blebs and fragmented nucleus. The images were obtained by an EVOS M5000 microscope (Thermo Fisher, Waltham, MA, USA). Representative images from three independent experiments are shown. Magnification of 20×.

The apoptotic cells exhibited characteristic morphological features of apoptosis, including the formation of apoptotic bodies, increased intercellular spacing, reduced cell volume, chromatin condensation, cell rounding, nuclear pyknosis, and plasma membrane blebbing. Compared to the control group, cells treated with all compounds at 5 μM for 24 h showed a markedly higher number of apoptotic cells, as illustrated in Figure 3. Red arrows in the figure highlight the presence of apoptotic bodies.

### 2.5. Cell Migration Quantified by the Cell-Based Scratch Assay

**Figure 4:**
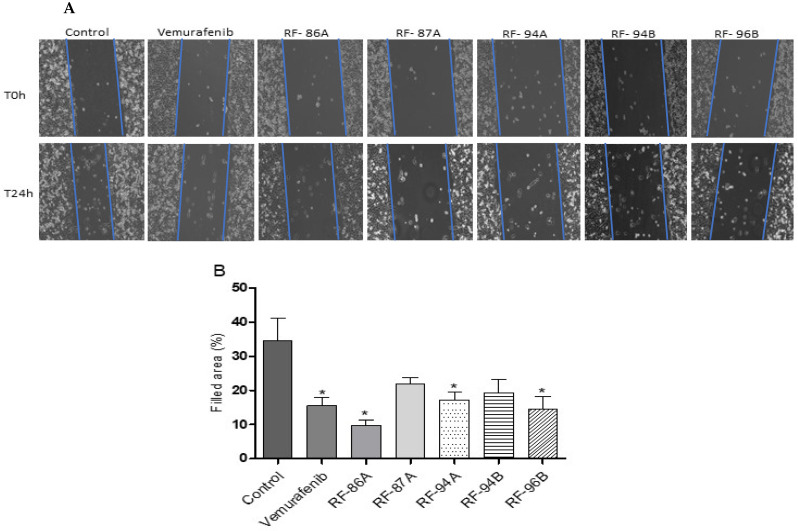
Effects of vemurafenib and analogs (**2a**–**e**) on migration of A375 cells. After incubation with medium alone (controls), and the treatment with the compounds test (0.5 μM) for 24 h, cell migration was assayed through cell-based scratch method. (**A**) The images were obtained by EVOS M5000 microscope (Thermo Fisher, Waltham, MA, USA). Representative images from three independent experiments are shown. Magnification of 10×. Scale bar 50 μm. (**B**) The filled areas were analyzed and quantified using ImageJ software (version 1.54p). Statistical analyses were performed with GraphPad Prism 8.02 and included one-way ANOVA followed by Dunnett’s post hoc test. Statistical significance was defined as * *p* < 0.05 when compared with the control group.

To assess whether vemurafenib and its analogs affect the migratory ability of A375 cells, we conducted a wound healing (scratch) assay followed by quantification of the wound area. Cells were grown to 90–100% confluence, and scratch wounds were created using a micropipette tip. Subsequently, cells were treated with 0.5 μM of vemurafenib or its analogs for 24 h. This concentration was selected to minimize interference with cell viability. Untreated cells served as the control group. Interestingly, after 24 h, untreated cells nearly closed the wound area, while cells treated with vemurafenib or any of the analogs retained significantly larger wound gaps (Figure 4A). The remaining wound areas in treated groups ranged from 25% to 50% compared to the fully closed wounds observed in control cultures (Figure 4B).

### 2.6. Effects of Vemurafenib and Analogs on A375 Cell Secretion of MMP-2 and MMP-9

**Figure 5:**
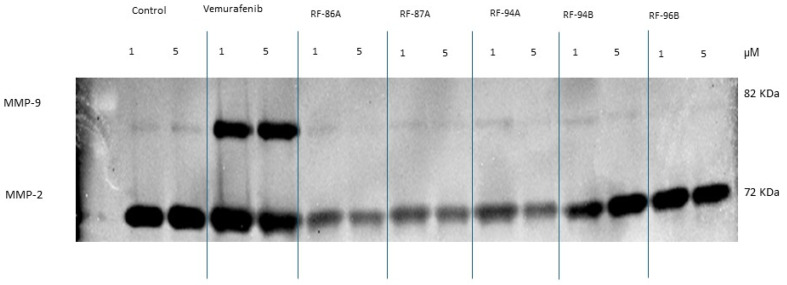
Effects of vemurafenib and analogs (**2a**–**e**) on MMP-9 and MMP-2 expression and activity by A375 cells after incubation with medium alone (controls), and the treatment with the test compounds (1 and 5 μm) for 24 h. Gelatinase activity was assayed through zymography method. Representative images from three independent experiments are shown. The protein bands were analyzed using 6.1 software from Bio-Rad.

The potential effects of vemurafenib and its analogs on MMP-2 and MMP-9 activity in A375 cells were evaluated using gelatin zymography to detect gelatinolytic activity in conditioned media. As shown in Figure 5, treatment of A375 cells for 24 h with 1 μM and 5 μM of vemurafenib or its analogs revealed that MMP-2 expression and activity were reduced following treatment with RF-86A (**2a**), RF-87A (**2b**), and RF-94A (**2c**). Notably, RF-87A reduced MMP-2 activity only at the lower concentration (1 μM). In contrast, RF-96B (**2e**) and vemurafenib did not reduce MMP-2 expression or activity at either concentration.

On the other hand, MMP-9 expression and activity were upregulated in response to vemurafenib treatment at both concentrations. This effect, however, was not observed with any of the analogs tested, suggesting a potential advantage of the analogs in limiting pro-metastatic MMP-9 activation.

## 3. Discussion

In this study, we synthesized, characterized, and evaluated five novel vemurafenib analogs designed to enhance the therapeutic profile by potentially reducing metastasis, more effectively eradicating tumor cells, and minimizing side effects.

We describe the rational design, chemical synthesis, and in vitro pharmacological evaluation of these novel vemurafenib analogs. We present evidence of the cytotoxic, anti-invasive, and anti-metastatic activities of the novel compounds against human melanoma cells. Our primary aim was to evaluate the initial biological effects of these molecules; future studies will focus on elucidating their molecular targets and the signaling pathways involved. This study specifically examined the effects of vemurafenib and its analogs on A375 melanoma cells, a well-established human cell line harboring the BRAFV600E mutation. In parallel, the non-tumor HEK293T cell line was used to assess potential off-target effects unrelated to the BRAFV600E mutation. Although the cancer-specific signaling pathways targeted by vemurafenib and its analogs may not be active or relevant in normal cells—thus limiting the interpretability of direct comparisons—the inclusion of HEK293T cells was essential for calculating the selectivity index of the tested compounds. This approach enabled the identification of RF-86A as the most promising candidate, exhibiting an IC_50_ value comparable to vemurafenib and the highest selectivity index among the series (Table 1).

For the final products (**2a**–**e**), it was possible to state that *N*-acylhydrazone (NAH) was formed through the presence of two characteristic signals in the ^1^H-NMR spectrum: amidic hydrogen (RCONH-CH=) as a broad signal with integral 1 by around 12 ppm, and the imine hydrogen (RCONH-CH=) as a simplet with integral 1 at approximately 8.5 ppm. In the ^13^C-NMR spectra, the two carbons of the NAH group were visualized at approximately 160 ppm (RCONH-CH=) and around 144 ppm (RCONH-CH=), confirming that the molecules were obtained.

In this scenario, it is worth highlighting that despite NAH being able to present two different diastereoisomers: the *E*-diastereoisomer and the *Z*-diastereoisomer [14], only the *E*-diastereoisomer was formed in four molecules (**2a**–**d**) in this series. We can strongly suggest the stereoselective formation of the reaction due to the absence of duplication of signals in all 1H-NMR spectra of the molecules in this series. This fact is already well described in the literature by our research group and other researchers [14,15,16].

However, molecule **2e** (RF-96B) was formed as a mixture of *E*/*Z* diastereoisomers since some specific signals close to the double bond were seen duplicated in NMR-^1^H. These signals were the imine hydrogen (8.27 and 8.11 ppm, integral = 1) and amidic hydrogen (11.17 and 11.05 ppm, integral = 1) and the methylene group (2.83 and 2.26 ppm, integral = 2) neighboring the carbonyl of the NAH fragment. In ^13^C-NMR, all signals referring to methylene carbons are duplicated. The formation of this mixture of diastereoisomers in NAH is due to the large conformational degree of freedom in hydrazide (**5e**). It has already been reported in the literature that hydrazides containing hybridized carbons such as sp^3^ near the carbonyl will form diastereoisomeric mixtures in NAH [11]. However, it should be noted that the ^1^H-NMR and ^13^C-NMR spectra of the methyl ester (**4e**) and hydrazide (**5e**) (Supplementary Materials) do not present duplicate signals, since there is no possibility of forming diastereoisomers.

This stereochemical complexity must be considered when evaluating the biological performance of such compounds, particularly in light of vemurafenib’s clinical limitations and the ongoing search for improved analogs. Vemurafenib is an FDA-approved chemotherapy drug widely used for the treatment of metastatic malignant melanoma, especially in patients harboring the BRAF^V600E^ mutation, where it induces significant tumor regression [17]. Nonetheless, this therapeutic response is frequently short-lived, as most patients eventually develop acquired resistance, resulting in tumor progression. Such limitations underscore the urgent need for alternative or optimized therapeutic strategies.

Such limitations underscore the urgent need for alternative or optimized therapeutic strategies capable of overcoming resistance mechanisms and sustaining clinical efficacy. In light of these therapeutic challenges, our study focused on the rational development and biological assessment of novel vemurafenib analogs aimed at enhancing efficacy and overcoming resistance.

Our results demonstrated that the analog compounds exerted cytotoxic effects on A375 human melanoma cells, inducing both morphological and molecular features consistent with apoptosis. Among them, compound RF-86A showed the highest potency, with an IC_50_ value more than threefold lower than those of the other analogs, and comparable to that of vemurafenib (6.99 vs. 7.68 μM, respectively). In contrast, vemurafenib exhibited no cytotoxicity toward HEK293T cells, resulting in a selectivity index greater than 1000. Notably, RF-86A also showed the most favorable selectivity index among the analogs, highlighting it as the most promising candidate in the series (Table 1).

A particularly noteworthy finding was the superior ability of the analogs to inhibit the activity and expression of matrix metalloproteinases (MMPs), specifically MMP-2 and MMP-9. These enzymes degrade extracellular matrix (ECM) components and basement membranes, facilitating tumor cell migration, invasion, metastasis, and angiogenesis. Elevated MMP expression correlates strongly with tumor progression and poor prognosis in malignant melanoma [18].

Both vemurafenib and the analogs effectively inhibited A375 cell migration in vitro. However, only the analogs significantly suppressed MMP-2 and MMP-9 activity, whereas vemurafenib did not reduce their expression or activity. This distinction is critical, as proteolytic remodeling of the ECM by MMPs is essential for metastatic dissemination.

MMP-9 serves as an established marker of melanoma invasiveness [19], and MMP-2 functions as a pro-metastatic factor during melanoma progression [20]. Increased expression of these MMPs has been documented not only in tumor cells but also in the surrounding stromal cells in vivo [21,22]. Our findings that the analogs suppress MMP-2 and MMP-9 after 24 h suggest that these compounds may provide a longer-lasting anti-metastatic effect compared to vemurafenib, potentially reducing metastatic potential in clinical contexts.

## 4. Materials and Methods

### 4.1. Chemistry

Reagents and solvents were purchased from suppliers and utilized without prior treatment. NMR spectra were acquired on a Bruker AVIII500 (11.7 T) operating at 500 MHz and on a Bruker AVHD400 (9.4 T; Billerica, MA, USA) operating at 400 MHz. Chemical shifts (δ) were reported in parts per million (ppm) relative to tetramethylsilane (TMS) as an internal standard, and coupling constant (*J*) values were expressed in Hertz (Hz). Deuterated solvent dimethyl sulfoxide-d6 (DMSO-*d_6_*) was employed to obtain the spectra, utilizing specific NMR glass tubes. Peak areas in the NMR spectra were determined by electronic integration, and multiplicities were denoted as follows: s (singlet), bs (broad signal), d (doublet), t (triplet), m (multiplet), dd (double doublet), and td (triple doublet). Infrared spectra were generated using the Varian FT-IR 660 Spectrometer (Agilent Technologies, Santa Clara, CA, USA).

The purity of the final products was determined by High-Performance Liquid Chromatography (HPLC) using a Shimadzu LC-20AD system equipped with a Kromasil 100-5 C18 column (250 × 4.6 mm, 5 µm) and an SPD-M20A diode array detector (Agilent Technologies, Santa Clara, CA, USA. The analyses were carried out at a detection wavelength of 300 nm, with a total runtime of 20 min. The mobile phase consisted of 80% acetonitrile in water under isocratic conditions. Purity was evaluated based on peak area normalization.

Previous studies have carefully described the synthetic methodologies used to prepare methyl esters (**3a**–**e**) in a published paper [23,24,25,26]. Similarly, hydrazides (**4a**–**e**) were synthesized using methods described in the literature [24,25,26,27]. Additionally, 5-bromo-1*H*-pyrrolo [2,3-*b*]pyridine-3-carbaldehyde (***7***) was synthesized as described by [13].

### 4.2. General Mechanism for Obtaining N-Acylhydrazones (***2a**–**e***)

To a 100 mL round-bottom flask, picolinohydrazide (**5d**, 0.18 g, 1.3 mmol, 1 equivalent), 5-bromo-1*H*-pyrrolo[2,3-*b*]pyridine-3-carbaldehyde (**7**, 0.35 g, 1.6 mmol, 1.2 equivalents), and 30 mL of ethanol were added. After turning on the magnetic stirring, 5 drops of hydrochloric acid were added to the reaction mixture. The reaction was then stirred for 6 h at room temperature, with precipitates forming during the first minutes of the reaction. After this time, the volume of ethanol was reduced under reduced pressure, and the solid was isolated through filtration using a porcelain funnel to obtain the desired final product (**2d**) (Figure S1 Supplementary Materials).

#### 4.2.1. (E)-N-((5-Bromo-1H-pyrrolo[2,3-b]pyridin-3-yl)methylene)-5,6,7,8-tetrahydronaphthalene-2-carbohydrazide (**2a** or RF-86B)

Pinkish amorphous solid; 70%; mp 260–261 °C. Purity (HPLC): 97.1%

IR (cm^−1^): 626 (ʋ C-Br), 1613 (ʋ C=C), 1651 (ʋ C=O), 3102 (ʋ N-H). ^1^H-NMR (500 MHz, DMSO) δ 12.34 (s, 1H, H7), 11.58 (s, 1H, H13), 8.74 (d, *J* = 2.2 Hz, 1H, H2), 8.56 (s, 1H, H11), 8.39 (d, *J* = 2.3 Hz, 1H, H6), 8.04 (d, *J* = 2.7 Hz, 1H, H17), 7.64 (d, *J* = 4.2 Hz, 2H, H8, H21), 7.21–7.17 (m, 1H, H18), 2.81–2.76 (m, 4H, H22, H25), 1.79–1.74 (m, 4H, H23, H24). ^13^C-NMR (126 MHz, DMSO) δ 162.6 (C14), 147.7 (C4), 144.0 (C11), 143.3 (C2), 140.6 (C20), 136.7 (C19), 131.9 (C8), 131.8 (C6), 130.8 (C16), 128.9 (C21), 128.0 (C18), 124.6 (C17), 118.3 (C5), 112.0 (C9), 110.4 (C1), 28.83 (C25) 28.81 (C22) 22.4 (C23), 22.5 (C24).

#### 4.2.2. (E)-N-((5-Bromo-1H-pyrrolo[2,3-b]pyridin-3-yl)methylene)picolinohydrazide (**2b** or RF-87A)

Light yellow amorphous solid; 91%; mp 245–246 °C. Purity (HPLC): 98.6%

IR (cm^−1^): 619 (ʋ C-Br), 1615 (ʋ C=C), 1671 (ʋ C=O), 3099 (ʋ N-H). ^1^H-NMR (500 MHz, DMSO) δ 12.36 (s, 1H, H7), 12.04 (s, 1H, H13), 8.76 (d, *J* = 2.2 Hz, 1H, H20), 8.74 (s, 1H, H11), 8.71 (d, *J* = 4.6 Hz, 1H, H2), 8.40 (d, *J* = 2.3 Hz, 1H, H17), 8.13 (d, *J* = 7.8 Hz, 1H, 6H), 8.06 (t, *J* = 7.7 Hz, 1H, H18), 8.02 (d, *J* = 2.7 Hz, 1H, H8), 7.68–7.64 (m, 1H, H19). ^13^C-NMR (126 MHz, DMSO) δ 160.0 (C14), 149.9 (C16), 148.4 (C20), 147.7 (C4), 144.9 (C2), 144.1 (C11), 138.01 (C18), 132.0 (C8), 131.9 (C6) 126.8 (C19), 122.5 (C17), 118.4 (C5), 112.1 (C9), 110.3 (C1).

#### 4.2.3. (E)-N-((5-Bromo-1H-pyrrolo[2,3-b]pyridin-3-yl)methylene)pyrazine-2-carbohydrazide (**2c** or RF-94A)

Pinkish amorphous solid; 88%; mp 304–306 °C. Purity (HPLC): 98.2%

IR (cm^−1^): 673 (ʋ C-Br), 1611 (ʋ C=C), 1643 (ʋ C=O), 3085 (ʋ N-H). ^1^H-NMR (500 MHz, DMSO) δ 12.39 (s, 1H), 12.20 (s, 1H), 9.27 (d, *J* = 1.3 Hz, 1H), 8.92 (d, *J* = 2.4 Hz, 1H), 8.79 (dd, *J* = 2.3, 1.5 Hz, 1H), 8.76 (d, *J* = 2.2 Hz, 1H), 8.74 (s, 1H), 8.40 (d, *J* = 2.3 Hz, 1H), 8.06 (d, *J* = 2.8 Hz, 1H). ^13^C-NMR (126 MHz, DMSO) δ 159.1 (C14), 147.7 (C4), 147.6 (C20), 145.6 (C19), 144.9 (C16), 144.1 (C11), 144.0 (C17), 143.3 (C2), 132.4 (C8), 131.9 (C6), 118.4 (C5), 112.2 (C9), 110.2 (C1).

#### 4.2.4. (E)-N-((5-Bromo-1H-pyrrolo[2,3-b]pyridin-3-yl)methylene)quinoxaline-2-carbohydrazide (**2d** or RF-94B)

Yellow amorphous solid; 84%; mp 286–288 °C. Purity (HPLC): 98.5%

IR (cm^−1^): 628 (ʋ C-Br), 1610 (ʋ C=O), 1668 (ʋ C=C), 3099 (ʋ N-H). ^1^H-NMR (500 MHz, DMSO) δ 12.41 (s, 1H, H7), 12.27 (s, 1H, H13), 9.53 (s, 1H, H17), 8.80 (s, 1H, H11), 8.78 (d, *J* = 2.3 Hz, 1H, H2), 8.41 (d, *J* = 2.3 Hz, 1H, H6), 8.27 (dd, *J* = 6.1, 3.7 Hz, 1H, H22), 8.21 (dd, *J* = 6.7, 3.2 Hz, 1H, H25), 8.08 (d, *J* = 2.4 Hz, 1H, H8), 8.03–7.99 (m, 2H, H23, H24). ^13^C-NMR (126 MHz, DMSO) δ 159.5 (C14), 147.8 (C4), 145.9 (C11), 144.6 (C19), 144.2 (C2), 144.1 (C17), 143.0 (C16), 139.8 (C20), 132.6 (C8), 132.1 (C6), 132.0 (C23), 131.4 (C25), 129.5 (C24), 129.2 (C22), 118.4 (C5), 112.3 (C9), 110.2 (C1).

#### 4.2.5. (EZ)-N-((1H-Pyrrolo[2,3-b]pyridin-3-yl)methylene)-4-(1H-indol-3-yl)butanehydrazide (**2e** or RF-96B)

White amorphous solid; 75%; mp 218–220 °C. Purity (HPLC): 96.8%

IR (cm^−1^): 689 (ʋ C-Br), 1619 (ʋ C=C), 1655 (ʋ C=O), 3292 (ʋ N-H). ^1^H-NMR (400 MHz, DMSO) δ 12.27 (bs, 1H, NH indole), 11.17 (s) and 11.05 (s, 1H, H3), 10.7 (d, 1H, NH azaindole) 8.65 (d, *J* = 2.3 Hz) and 8.53 (d, *J* = 2.2 Hz, 1H, H10), 8.38 (dd, *J* = 5.9, 2.3 Hz, H20), 8.27 (s) and 8.11 (s, 1H, H5), 7.97 (d, *J* = 5.2 Hz, 1H, H13), 7.53 (d, *J* = 7.9 Hz, 1H, H11), 7.37–7.29 (m, 1H, H24), 7.14 (d, *J* = 5.8 Hz, 1H, H27), 7.09–6.88 (m, 2H, H25 and H26), 2.83 (t, *J* = 7.4 Hz) and 2.26 (t, *J* = 7.4 Hz, 2H, H1) 2.73 (dd, *J* = 18.2, 7.8 Hz, 2H, H18), 2.00 (td, *J* = 14.7, 7.6 Hz, 2H, H17). ^13^C-NMR (100 MHz, DMSO) δ 173.76 (C12), 168.2 (C2), 147.6 (C8), 143.9 (C5), 141.8 (C13), 138.7 (C10), 136.3 (C22), 131.8 (C11), 131.4 (C20), 127.2 (C23), 122.3 (C25), 120.8 (C26), 118.3 (C27), 114.1 (C7), 112.0 (C6), 111.3 (C24), 110.3 (C19), 34.0 and 32.5 (C1), 25.9 and 25.4 (C18), 24.7 and 24.3 (C17).

### 4.3. Chemicals

Vemurafenibe was obtained from MedChemExpress (Monmouth Junction, NJ, USA). Vemurafenib and vemurafenib analogs RF-86A (**2a**), RF-97A (**2b**), RF-94A (**2c**), RF-94B (**2b**), and RF-96B (**2e**) were dissolved in DMSO (dimethyl sulfoxide, Sigma-Aldrich, St. Louis, MO, USA) and further diluted in sterile culture medium immediately before their use. DMSO did not exceed 0.3% *v*/*v* in the culture medium.

### 4.4. Cell Culture

The human cutaneous melanoma cancer cell line A375, obtained from the American Type Culture Collection (ATCC, Rockville, MD, USA) and human embryonic kidney HEK293T cell line, obtained from Rio de Janeiro Cell Bank (BCRJ, Rio de Janeiro, Brazil), were cultured in Dulbecco’s Modified Eagle’s Medium (DMEM, Sigma-Aldrich, St. Louis, MO, USA) supplemented with L-glutamine (2 mM), 10% fetal bovine serum (FBS), 50 IU/mL penicillin and 50 μg/mL streptomycin (Sigma-Aldrich, St. Louis, MO, USA). Cells were maintained at 37 °C in a humidified atmosphere containing 5% CO_2_. Low cell passages (between 5 and 20) were used in the present study.

### 4.5. Cell Viability Assay

Cell viability was measured using an MTS method based on the conversion of the tetrazolium salt MTS (3-(4,5-dimethylthiazol-2-yl)-5-(3-carboxymethoxyphenyl)-2-(4-sulfophenyl)-2*H*-tetrazolium, Sigma-Aldrich, St. Louis, MO, USA) to a purple formazan in the presence of phenazine methosulfate. The enzymes responsible are NADPH-dependent dehydrogenases, which are active in viable cells. Briefly, cells (5 × 10^4^/well) were seeded in 96-well plates in medium with 10% FBS; after 24 h, the complete medium was replaced with fresh medium containing 1% FBS and incubated for 24 h. Fresh stock solutions were prepared on the day of the experiment, and the cells were treated with different concentrations of vemurafenib and analogs (0.01, 0.1, 0.5, 1, 5, and 10 μM). Effects were evaluated after 24 h. Untreated cells were incubated as controls. Following drug exposure, MTS was added, and the absorbance was measured at 570 nm using a microplate reader (CLARIOStar Plus, BMG LabTech, Ortenberg, Germany).

### 4.6. DNA Fragmentation Analyses

To detect DNA fragmentation in A375 cells, a modified apoptosis TUNEL method was used with the MEBSTAIN Apoptosis TUNEL Kit Direct (MBL International Corporation, Woburn, MA, USA) according to the manufacturer’s protocol. Briefly, cells were grown in 8-well culture slides (Corning, NY, USA) and incubated with the compounds (5 μM) for 24 h. Following treatments, the cells were washed with PBS and subsequently stained with TdT buffer, TdT solution, and Propidium Iodide, according to the manufacturer’s instructions. Experiments were conducted by fluorescent microscopy. The images were collected by an EVOS M5000 microscope (Thermo Fisher Scientific, Waltham, MA, USA).

### 4.7. Cell Morphology Analysis

In order to investigate cell apoptosis, morphological analysis was performed by contrast phase microscopy. Briefly, cells were grown in 8-well culture slides (Corning, Glendale, AZ, USA) and incubated with the compounds (5 μM) for 24 h. Following treatments, the cells were washed with PBS and subsequently fixed with PFA (paraformaldehyde) 4% solution, followed by observation under an EVOS M5000 microscope (Thermo Fisher Scientific, Waltham, MA, USA).

### 4.8. Cell Scratch Assay

Cellular migration was evaluated by scratch assay according to our previous studies [28,29]. Cells were cultured in 12-well culture plates for 24 h up to 90–100% confluence. The cells were incubated for 24 h with compound testing at final concentrations of 1 μM. All cell-based scratch assays were performed in the presence of the anti-mitotic reagent cytosine arabinoside (AraC; Sigma-Aldrich, St. Louis, MO, USA) at a final concentration of 10–5 M (to inhibit cell proliferation). After treatment with the compounds, the wound areas were observed, and images were acquired with an Evos M5000 (Invitrogen, Thermo Fischer Scientific Inc., Waltham, MA, USA). The filled area was quantified using the Fiji software (ImageJ, version 1.54p, National Institutes of Health, Bethesda, MD, USA).

### 4.9. Gelatin Zymography

Gelatin zymography was carried out according to [30], using 7.5% SDS-polyacrylamide gels with 0.2% (*w*/*v*) gelatin. SDS-PAGE was run at 100 V at room temperature. Following electrophoresis, the gels were removed and placed in 2.5% (*v*/*v*) Triton X-100 to renature enzymes for 60 min. Then, gels were incubated in 50 mM Tris-HCl, 1 μM ZnCl_2_, 5 mM CaCl_2_, pH 7.5, overnight, to allow sufficient time for genatinolytic activity to occur. Following overnight incubation at 37 °C, gels were incubated in a staining solution (0.5% (*v*/*v*) Coomassie blue in a 40% methanol-10% acetic acid-water mixture) for 60 min. Following incubation, the staining solution, the gels were washed with the destaining solution (without coomassie blue) until the time to start to see the bands. Then imaged with a ChemiDoc^TM^ MP (BIO-RAD, Hercules, CA, USA) gel imaging system. Total protein and gelatinase activity were quantified using Image Lab™ Software 6.1 (BIO-RAD, Hercules, CA, USA).

### 4.10. Statistical Analysis

All the values of in vitro experiments are presented as the mean ± standard error of three independent experiments. Statistical analyses were performed via a one-way ANOVA with Dunnett’s post hoc test, and statistical significance was defined as * *p* < 0.05. IC_50_ values were calculated by non-linear regression analysis using an Inhibitor vs. normalized response model curve fit, as implemented. Statistical analyses were performed with GraphPad Prism 10.1.2 (GraphPad Software Inc., San Diego, CA, USA).

## 5. Conclusions

Our findings demonstrate, for the first time, that five newly synthesized vemurafenib analogs—RF-86A, RF-87A, RF-94A, RF-94B, and RF-96B—effectively reduce the viability of A375 human melanoma cells and inhibit metastatic potential through suppression of MMP-2 and MMP-9 activity. Among them, RF-86A showed the most potent cytotoxic activity, with an IC_50_ more than three times lower than the other analogs, although vemurafenib still exhibited greater cytotoxicity overall. Notably, all analogs demonstrated superior inhibition of MMP activity compared to vemurafenib, indicating their potential for enhanced anti-metastatic effects. These results suggest that targeted structural modifications of vemurafenib may yield new therapeutic strategies with prolonged efficacy and reduced risk of metastasis. Future studies, including evaluation of selectivity in normal cells and in vivo models, are warranted to validate the therapeutic potential and safety profile of these novel analogs in the treatment of metastatic melanoma.

## Figures and Tables

**Table 1:** IC_50_ values and selectivity indices for vemurafenib and the analog compounds against A375 and HEK293T cell lines.

	A375		HEK293T		
Compound	IC_50_ (µM)	95% CI	IC_50_ (µM)	95% CI	Selectivity Index
Vemurafenib	7.68	5.334 to 11.15	>1000	-	>1000
RF-86A (**2a**)	6.99	4.864 to 10.15	114.4	43.47 to ∞	16.37
RF-87A (**2b**)	47.22	36.72 to 64.08	142.3	38.60 to ∞	3.01
RF-94A (**2c**)	53.01	39.29 to 77.77	25.76	22.62 to 29.61	0.49
RF-94B (**2d**)	46.33	35.81 to 63.40	25.52	15.79 to 48.59	0.55
RF-96B (**2e**)	21.32	15.54 to 30.84	14.32	9.572 to 22.76	0.67

Cells were incubated with different concentrations of each substance. Inhibitory concentrations that reduced in 50% cell viability (IC_50_) values were calculated using GraphPad Prism software (version 10.8.2). Selectivity index (SI) was calculated by dividing IC_50_ value obtained with non-tumoral cell (HEK293T) and tumoral cell (A375).

## Data Availability

All data are present in the manuscript.

## Supplementary Materials

The following supporting information can be downloaded at: https://www.mdpi.com/article/10.3390/ph18081161/s1, Figure S1: spectrum of each purification.
