# Supplementary material for: Design, Synthesis, and Bioactivity Assessment of Modified Vemurafenib Analog
Source: Pharmaceuticals (Basel). 2025 Aug 5;18(8):1161. doi: 10.3390/ph18081161 (PMC12389646; doi:10.3390/ph18081161)
Supplement: Supplementary file 1 [file pharmaceuticals-18-01161-s001.zip › pharmaceuticals-3659120-supplementary.pdf]

Design, synthesis and bioactivity assessment of modified vemurafenib analogs

Fabiana Sélos Guerra[1], Rosana H. C. N. Freitas[2,3], Florina Moldovan[4], David R. Rocha[2], Patricia Dias Fernandes[1]

[1] Laboratório de Farmacologia da Dor e da Inflamação, Instituto de Ciências Biomédicas, Universidade Federal do Rio de Janeiro, 21941-902, Rio de Janeiro, RJ, Brasil
[2] Laboratório de Síntese de Substâncias de Interesse Biológico (SiMIB), Universidade Federal Fluminense, Instituto de Química, Campus do Valonguinho, 24020-141, Niterói, RJ, Brasil
[3] Universidade Federal do Estado do Rio de Janeiro, Instituto de Química, Centro de Tecnologia, 20950-000, Rio de Janeiro, RJ, Brasil
[4] Centre Hospitalier Universitaire St. Justine, Université de Montréal, Montréal, QC, Canada

Supplementary Material

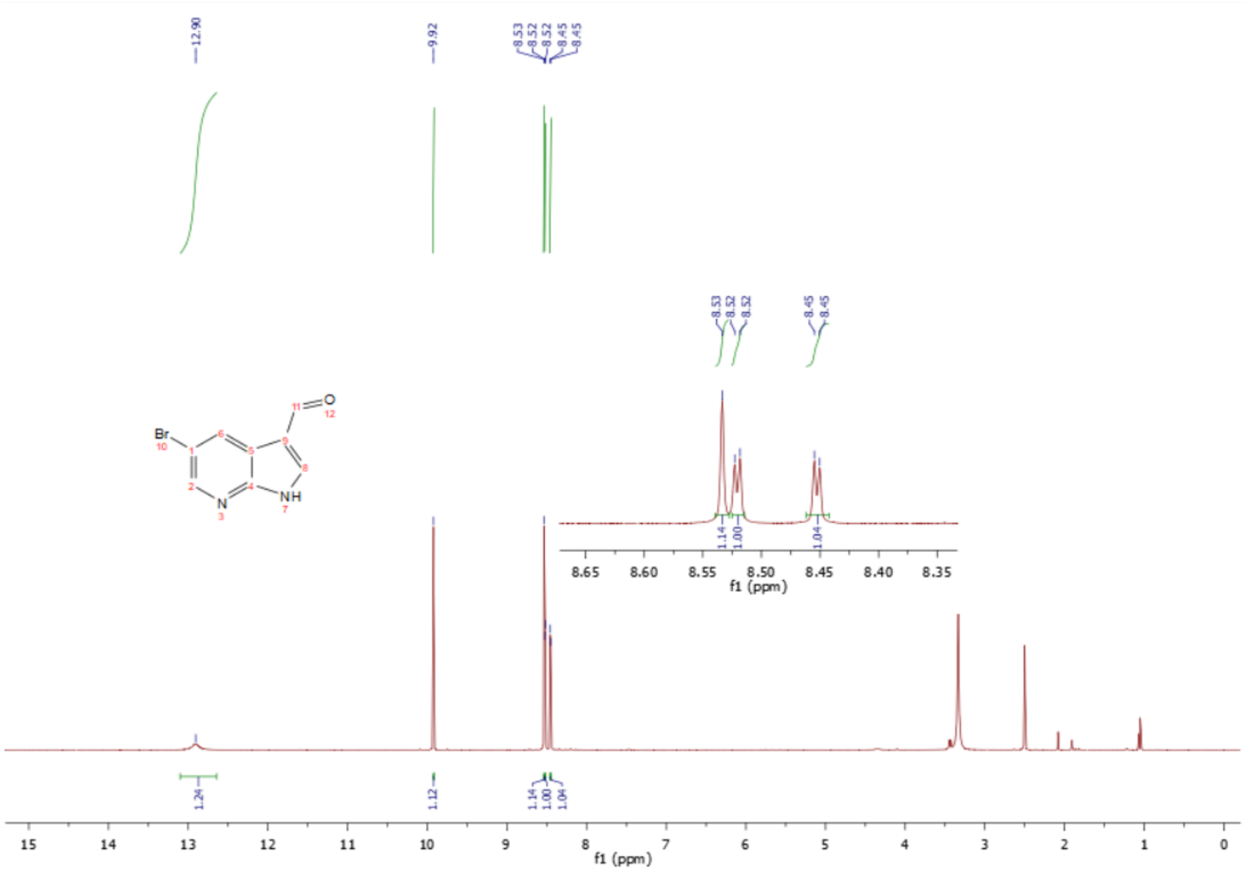

^{1}H-NMR of 5-bromo-1*H*-pyrrolo[2,3-*b*]pyridine-3-carbaldehyde (**7**) (DMSO-d_6, 500 MHz).

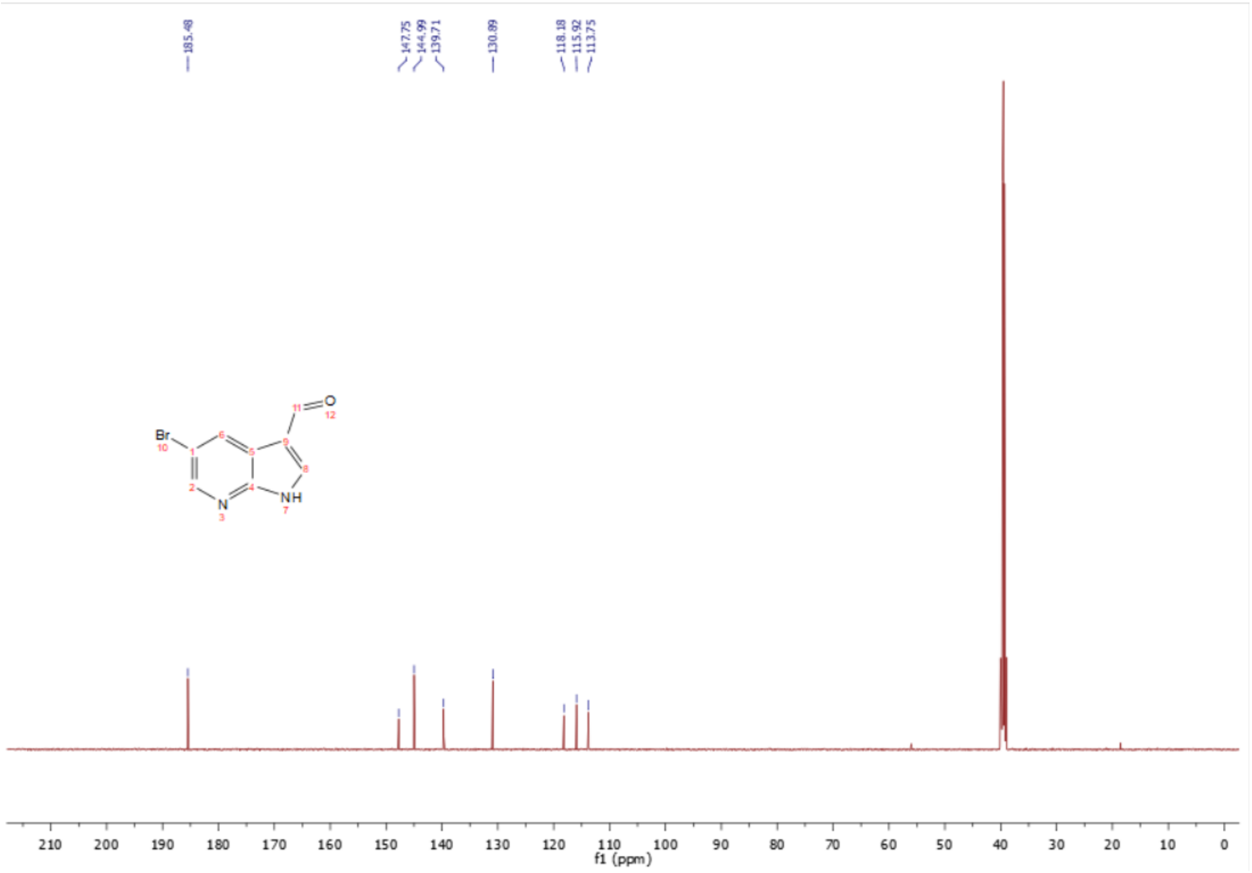

^{13}C-NMR of 5-bromo-1*H*-pyrrolo[2,3-*b*]pyridine-3-carbaldehyde (**7**) (DMSO-d_6, 125 MHz).

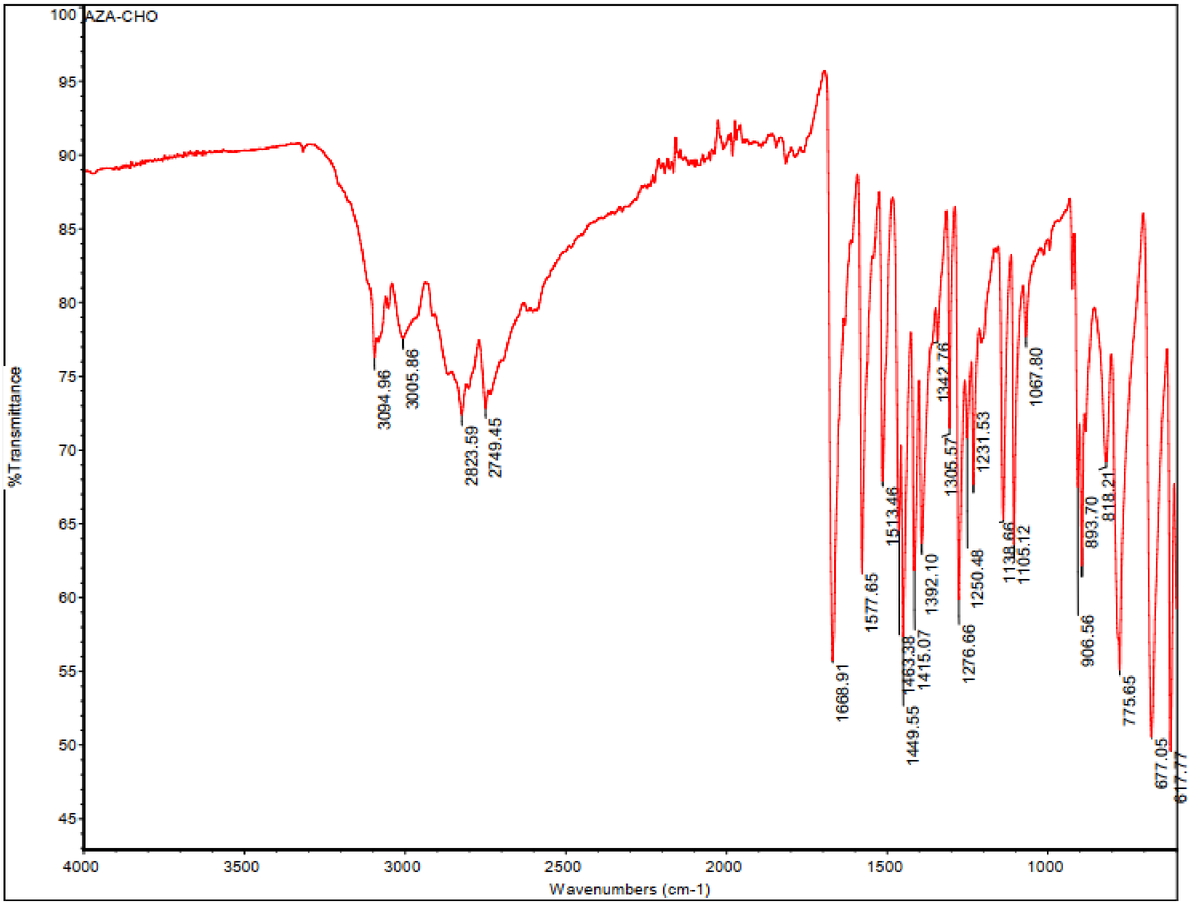

IR spectra of 5-bromo-1*H*-pyrrolo[2,3-*b*]pyridine-3-carbaldehyde (**7**) (cm^{-1})

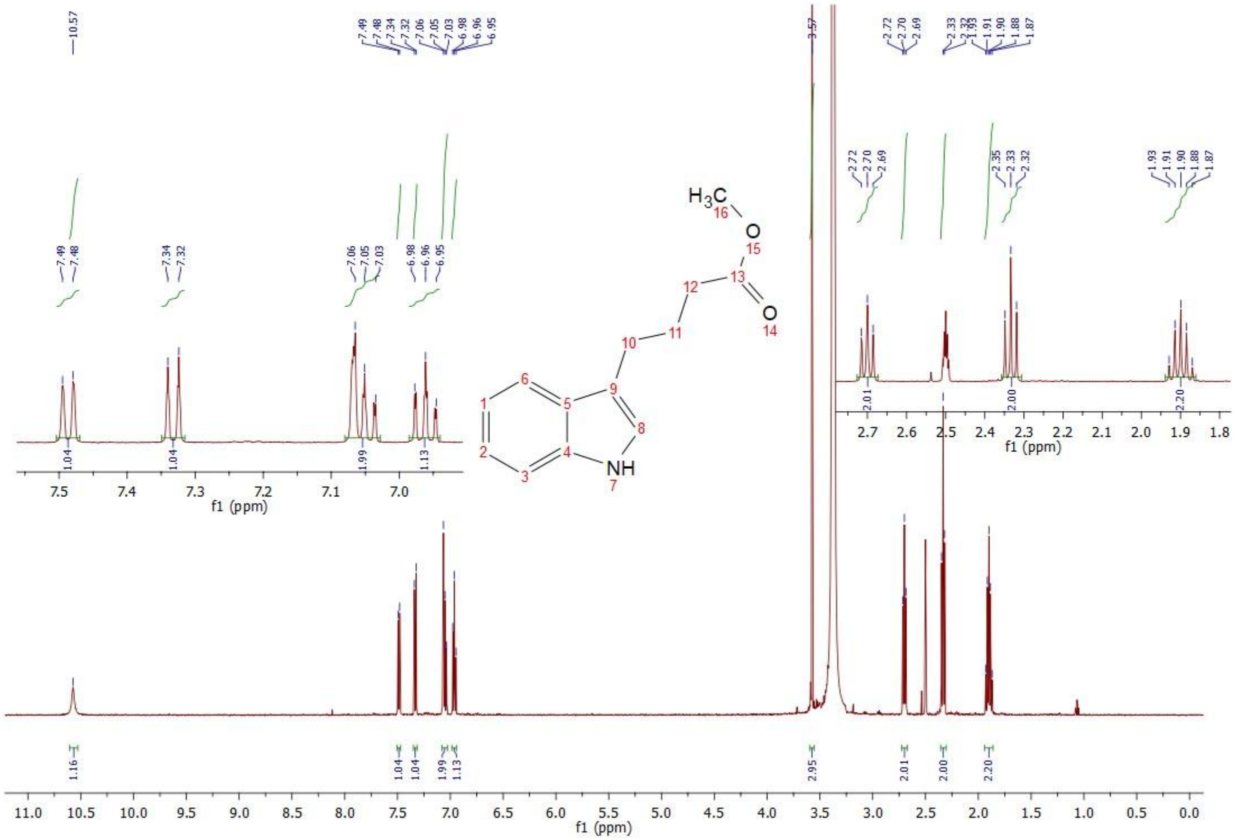

^{1}H-NMR of methyl 4-(1H-indol-3-yl)butanoate (**4e**) (DMSO-d_6, 500 MHz)

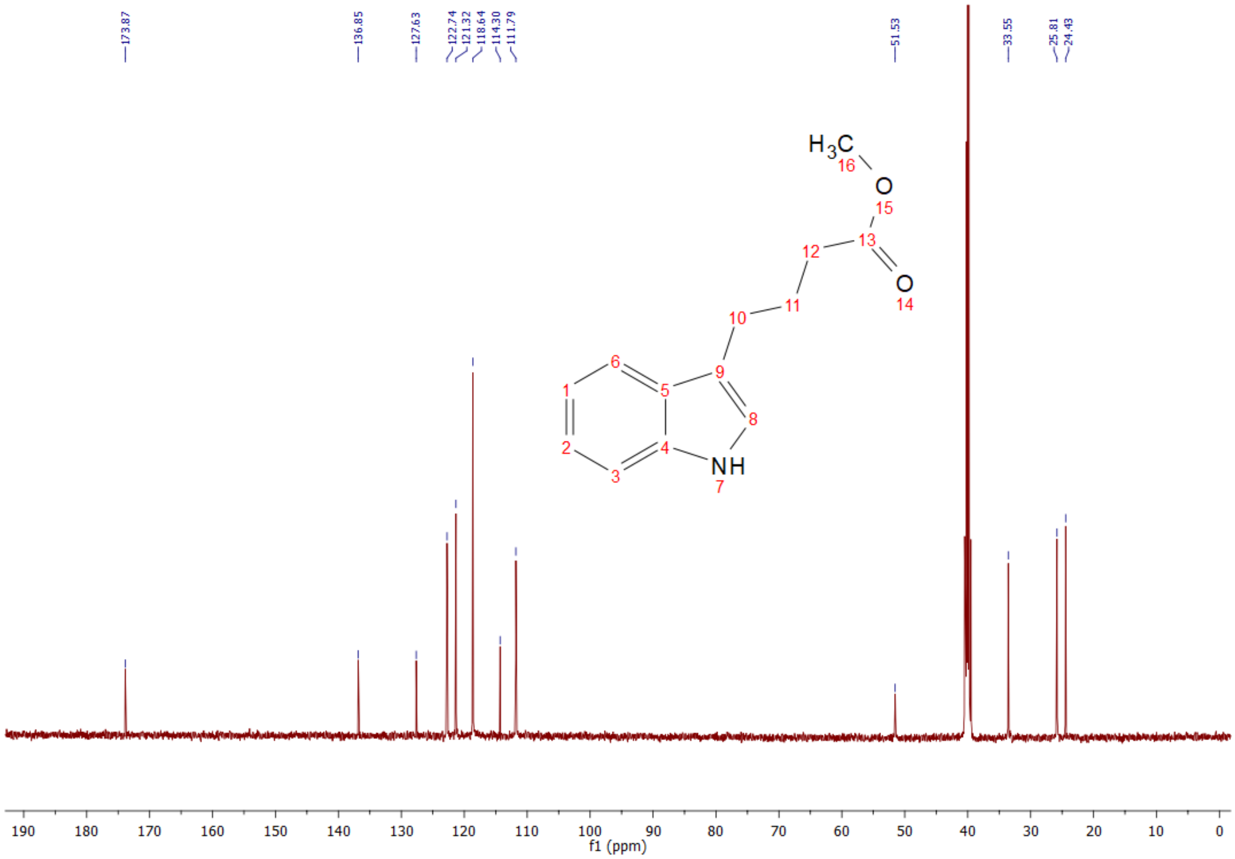

^{13}C-NMR of methyl 4-(1H-indol-3-yl)butanoate (**4e**) (DMSO-d_6, 125 MHz)

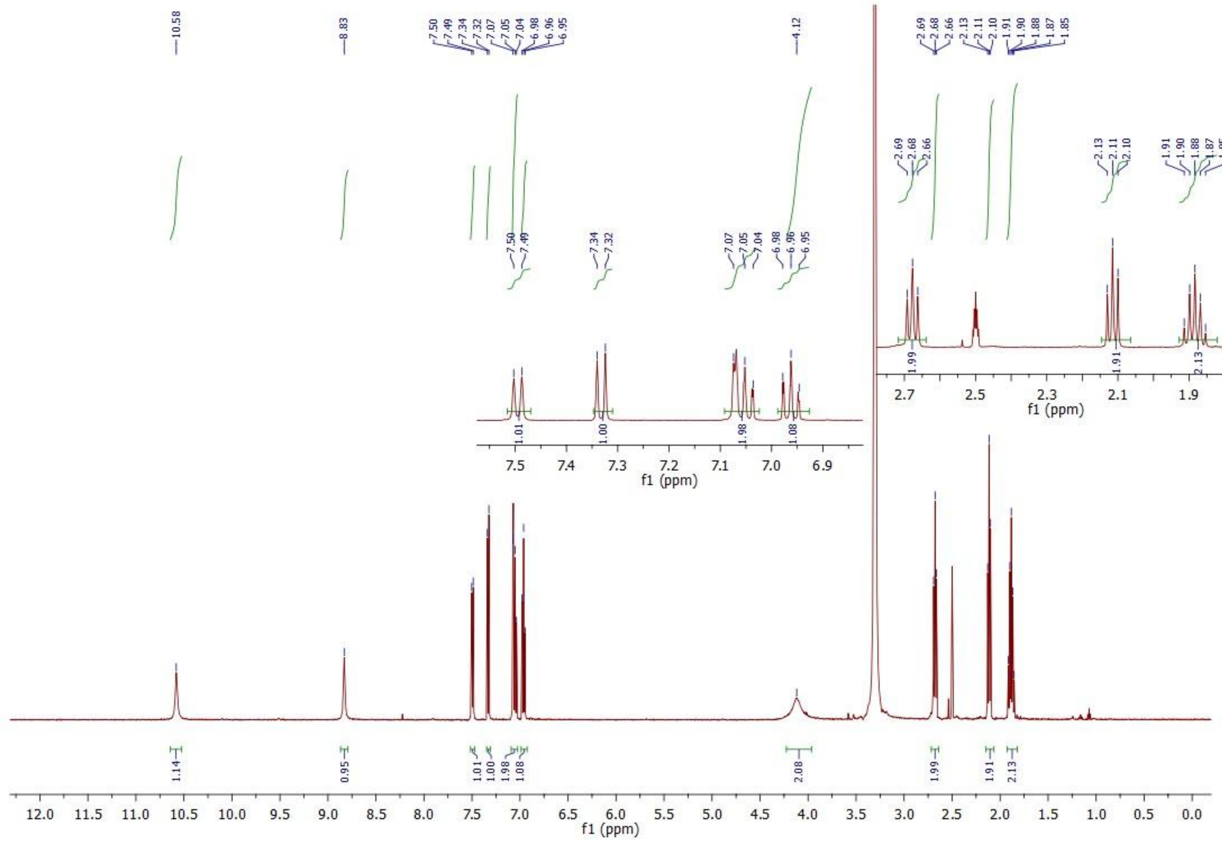

^{1}H-NMR of 4-(1H-indol-3-yl)butanehydrazide (**5e**) (DMSO-d_6, 500 MHz)

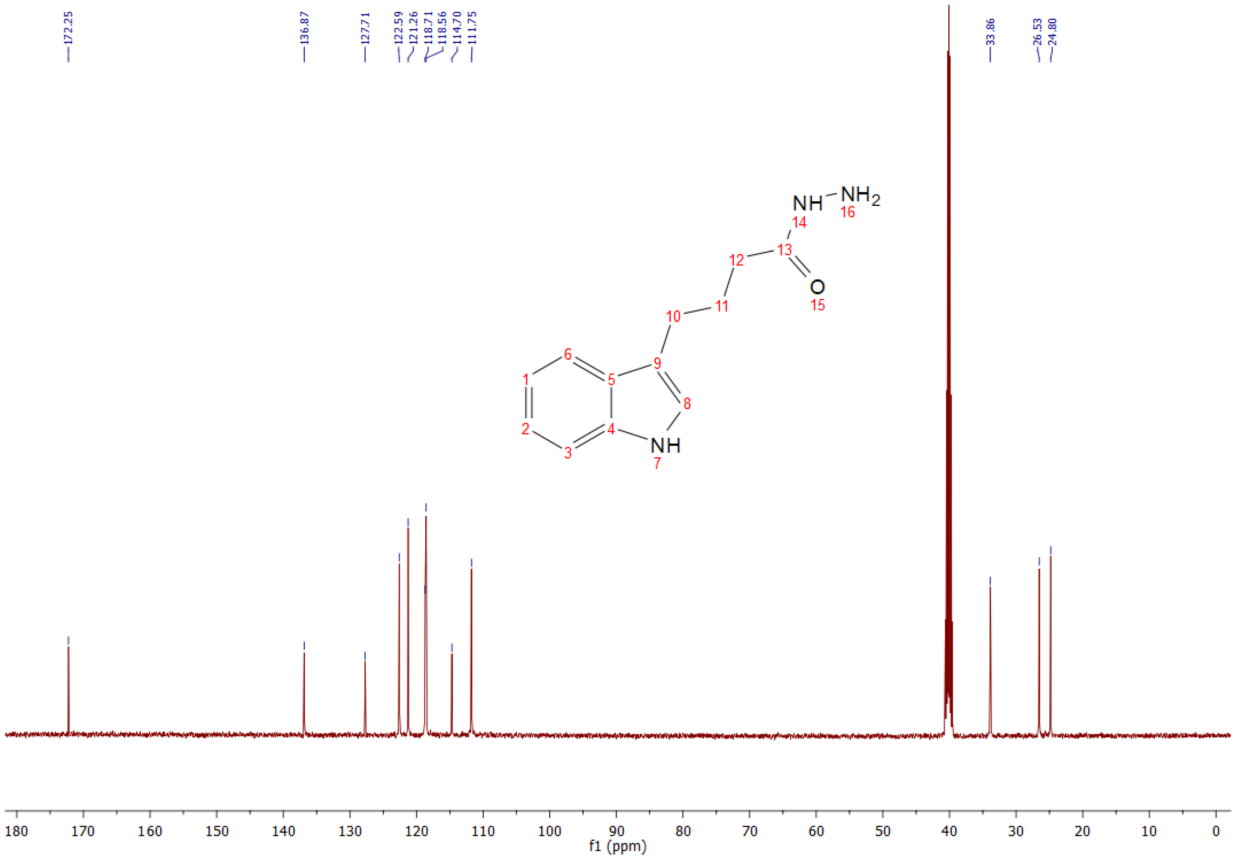

^{13}C-NMR of 4-(1H-indol-3-yl)butanehydrazide (**5e**) (DMSO-d_6, 125 MHz).

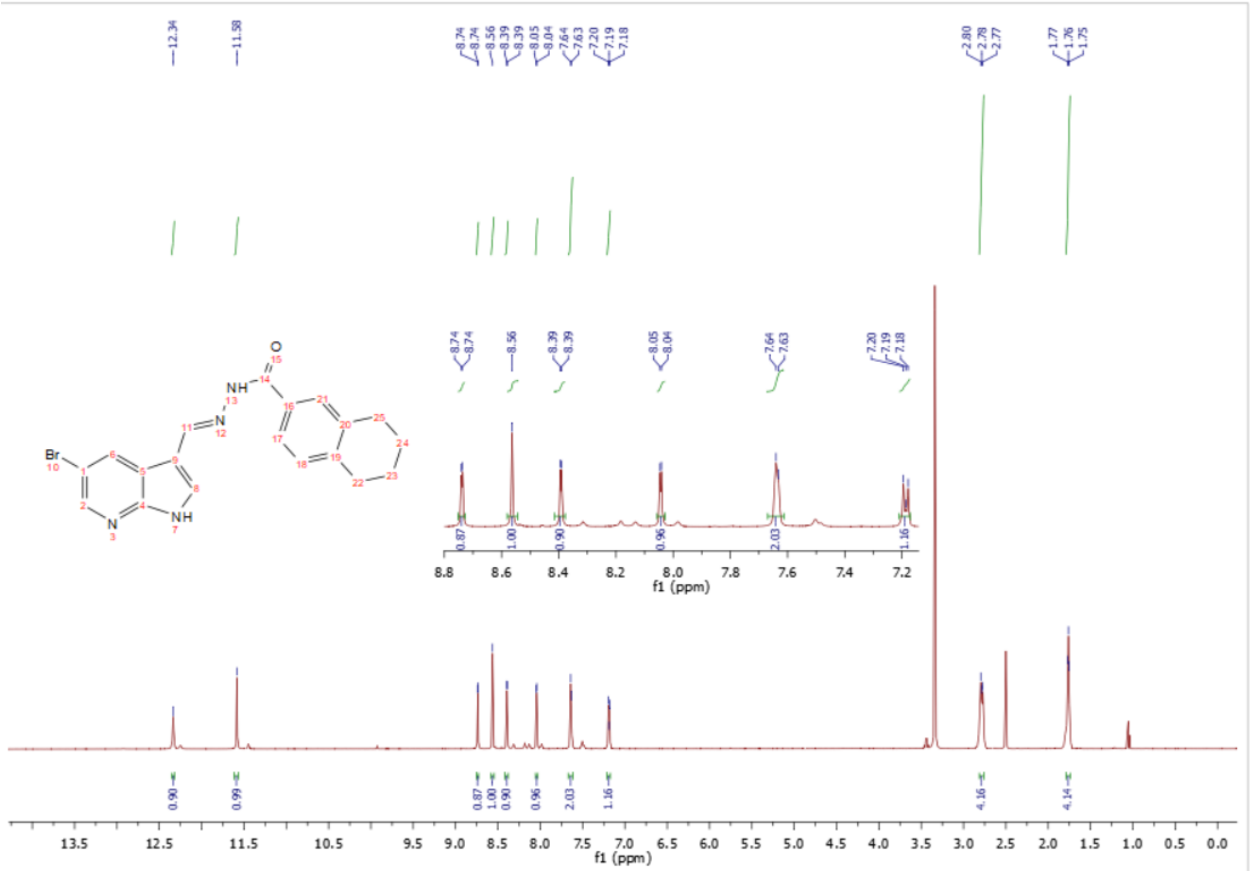

^{1}H-NMR of (*E*)-*N*'-((5-bromo-1*H*-pyrrolo[2,3-*b*]pyridin-3-yl)methylene)-5,6,7,8-tetrahydronaphthalene-2-carbohydrazide (**RF-86A** or **2a**) (DMSO-d_6, 500 MHz).

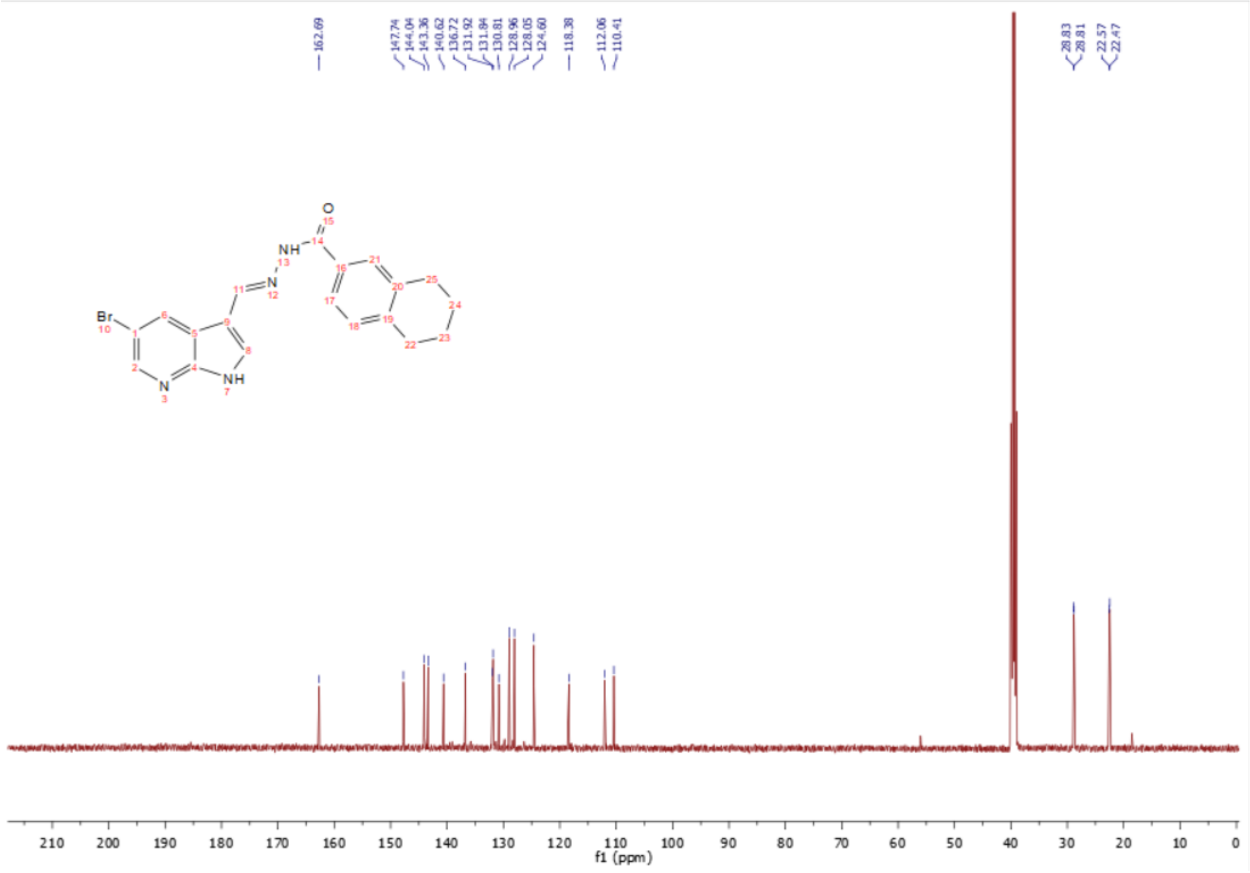

^{13}C-NMR of (*E*)-*N*'-((5-bromo-1*H*-pyrrolo[2,3-*b*]pyridin-3-yl)methylene)-5,6,7,8-tetrahydronaphthalene-2-carbohydrazide (**RF-86A** or **2a**) (DMSO-*d*$_6$, 125 MHz).

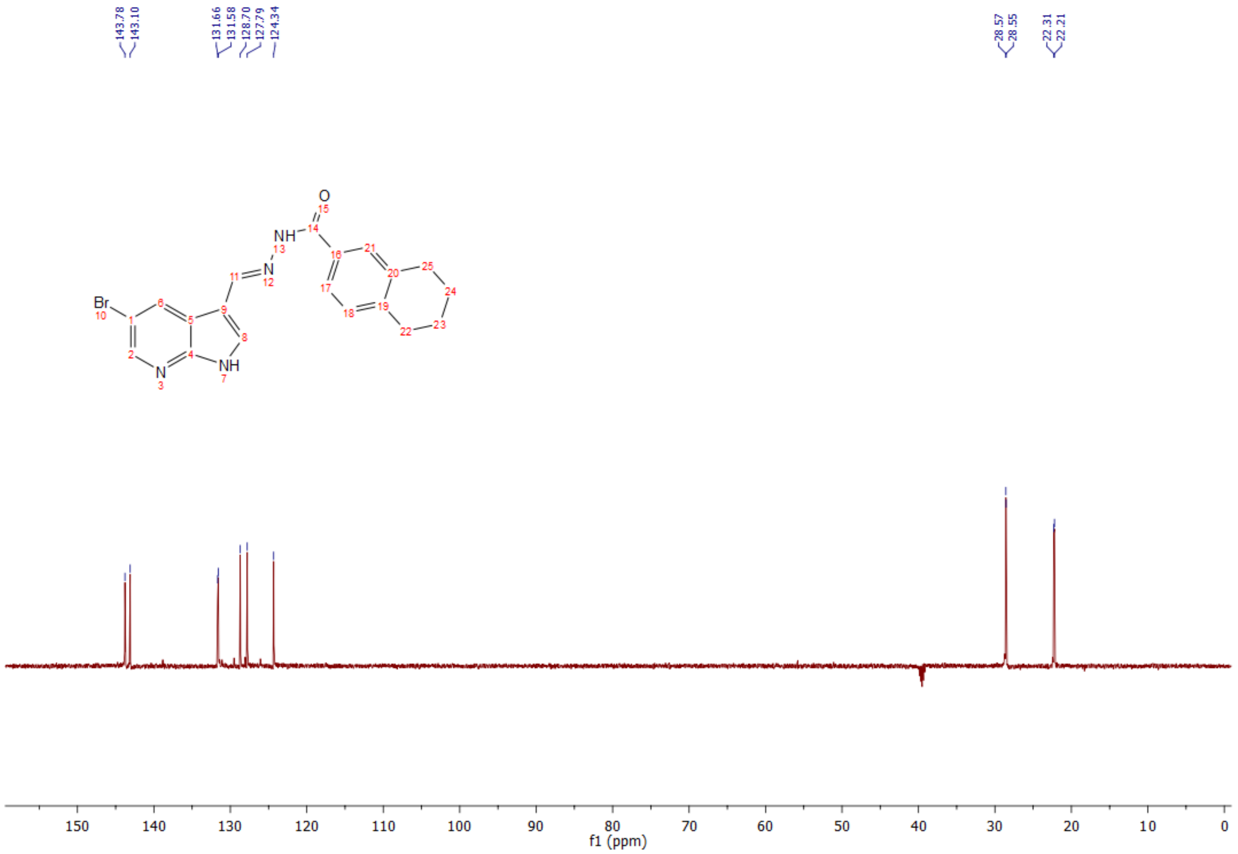

DEPT-135 experiment of(*E*)-*N*'-((5-bromo-1*H*-pyrrolo[2,3-*b*]pyridin-3-yl)methylene)-5,6,7,8-tetrahydronaphthalene-2-carbohydrazide (**RF-86A** or **2a**) (DMSO-d_6, 63 MHz).

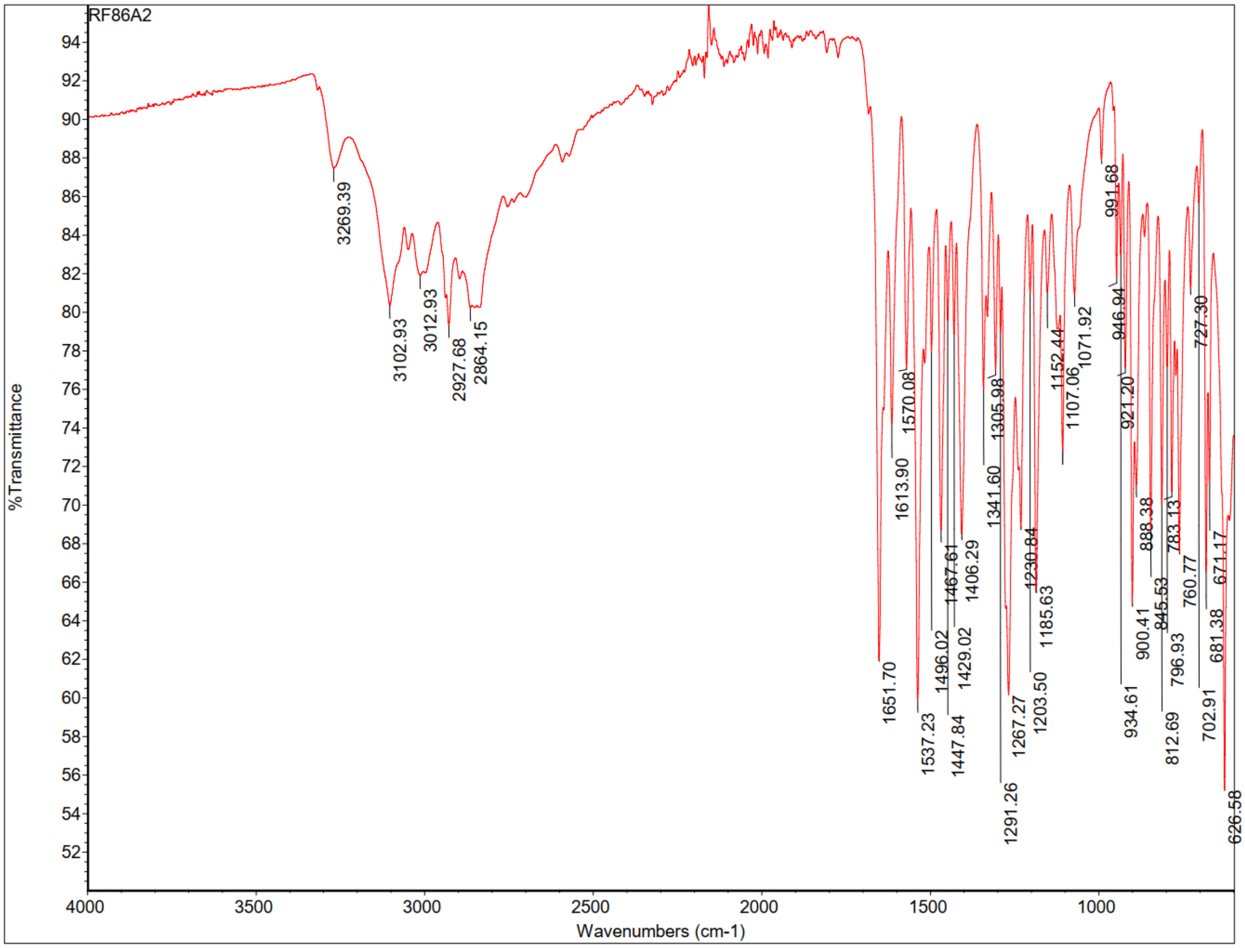

IR spectra of (*E*)-*N*'-((5-bromo-1*H*-pyrrolo[2,3-*b*]pyridin-3-yl)methylene)-5,6,7,8-tetrahydronaphthalene-2-carbohydrazide (**RF-86A** or **2a**) (cm^{-1}).

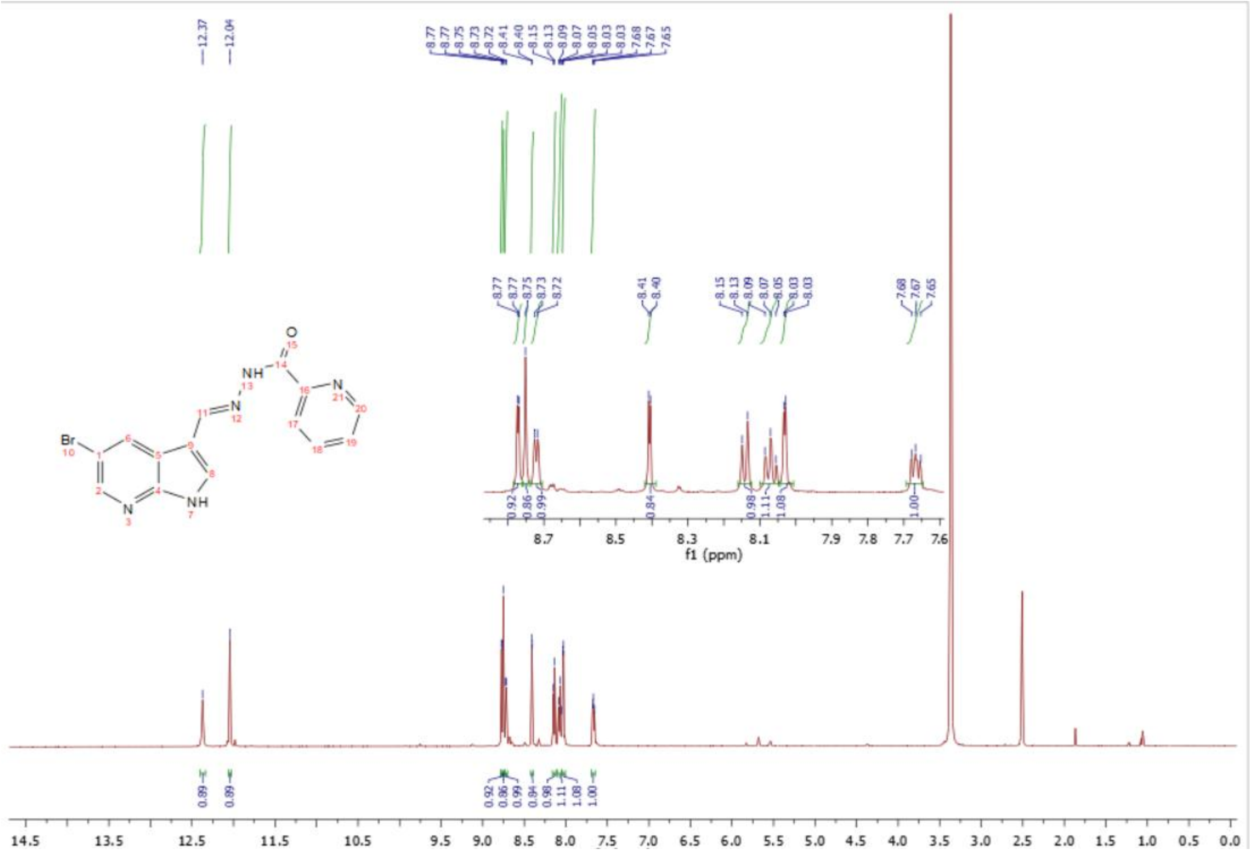

^{1}H-NMR of (*E*)-*N*'-((5-bromo-1*H*-pyrrolo[2,3-*b*]pyridin-3-yl)methylene)picolinohydrazide (**RF-87A** or **2b**) (DMSO-d_6, 500 MHz).

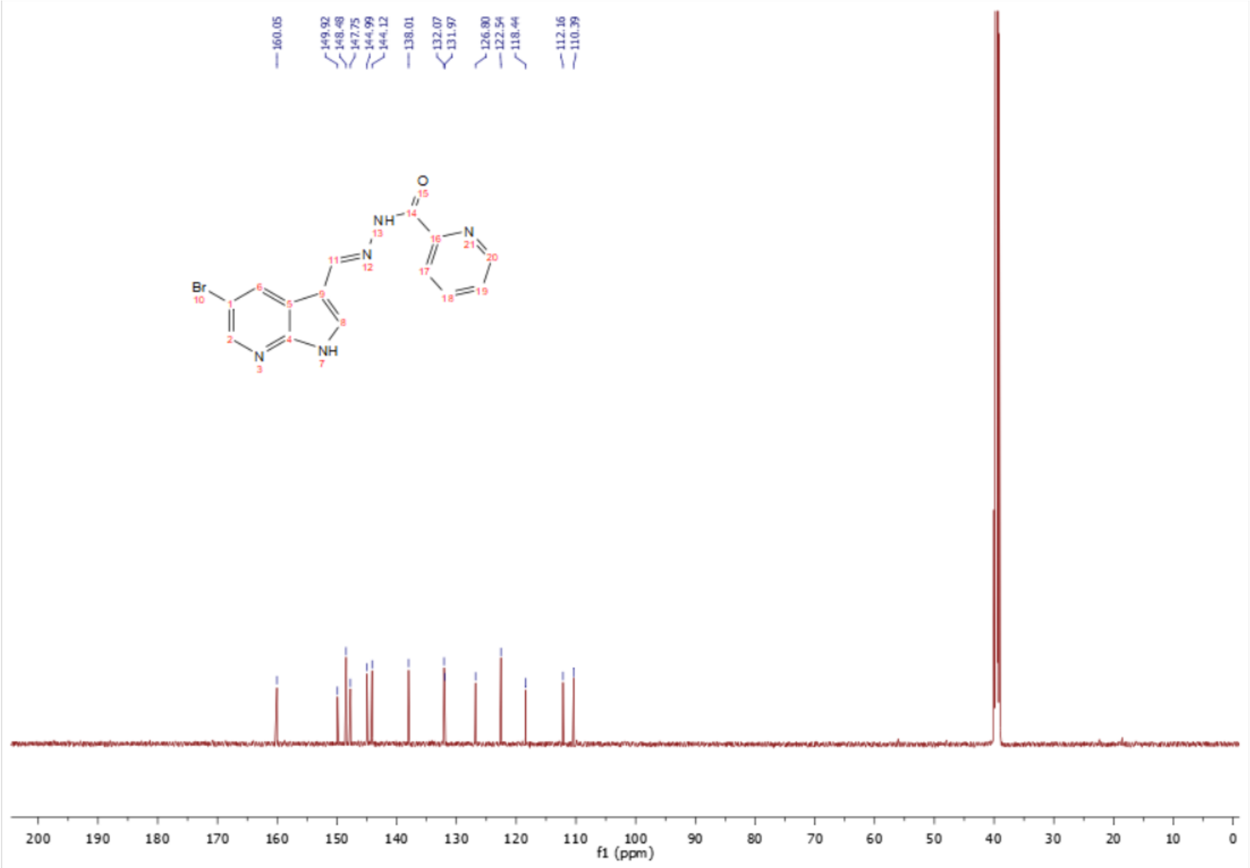

^{13}C-NMR of (*E*)-*N*'-((5-bromo-1*H*-pyrrolo[2,3-*b*]pyridin-3-yl)methylene)picolinohydrazide (**RF-87A** or **2b**) (DMSO-d_6, 125 MHz).

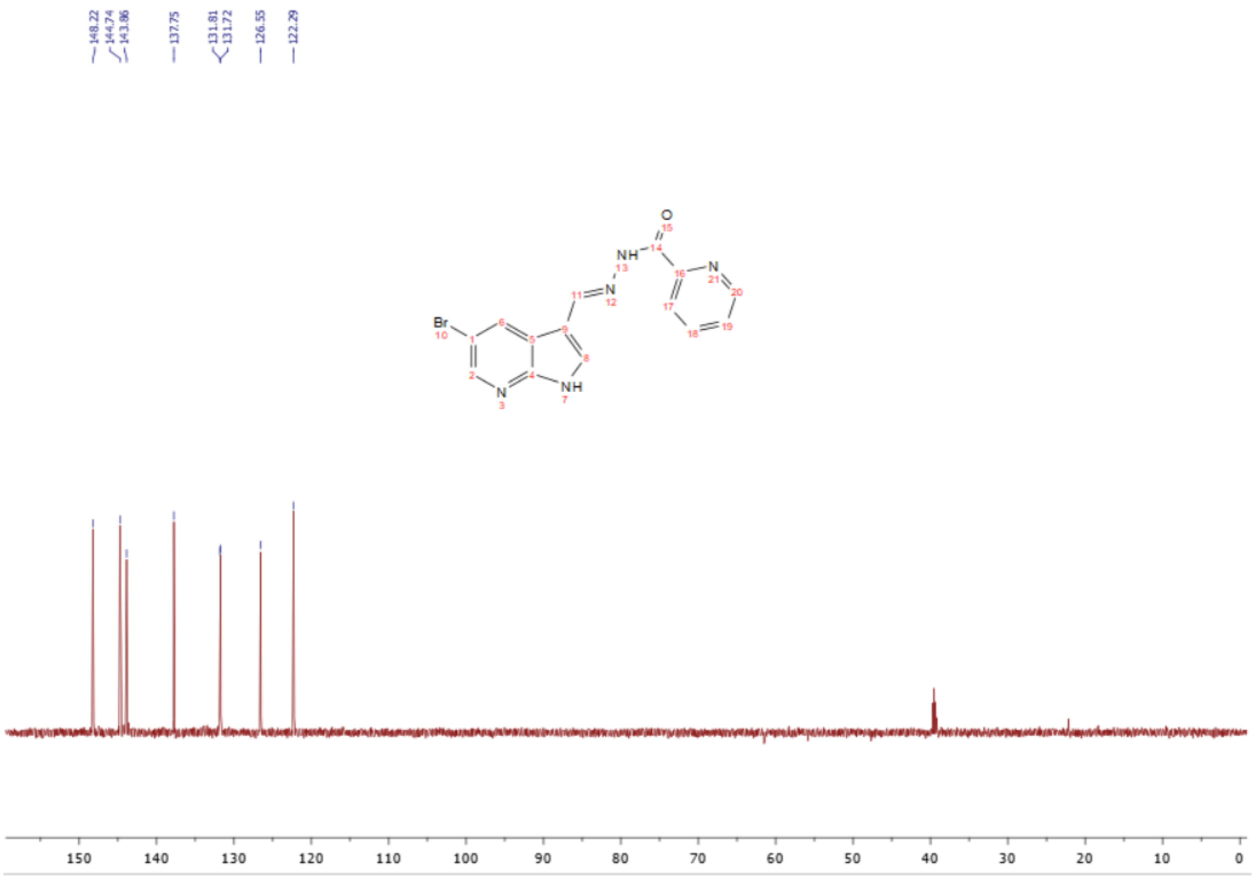

DEPT-135 experiment of (*E*)-*N*'-((5-bromo-1*H*-pyrrolo[2,3-*b*]pyridin-3-yl)methylene)picolinohydrazide (**RF-87A** or **2b**) (DMSO-d_6, 60 MHz).

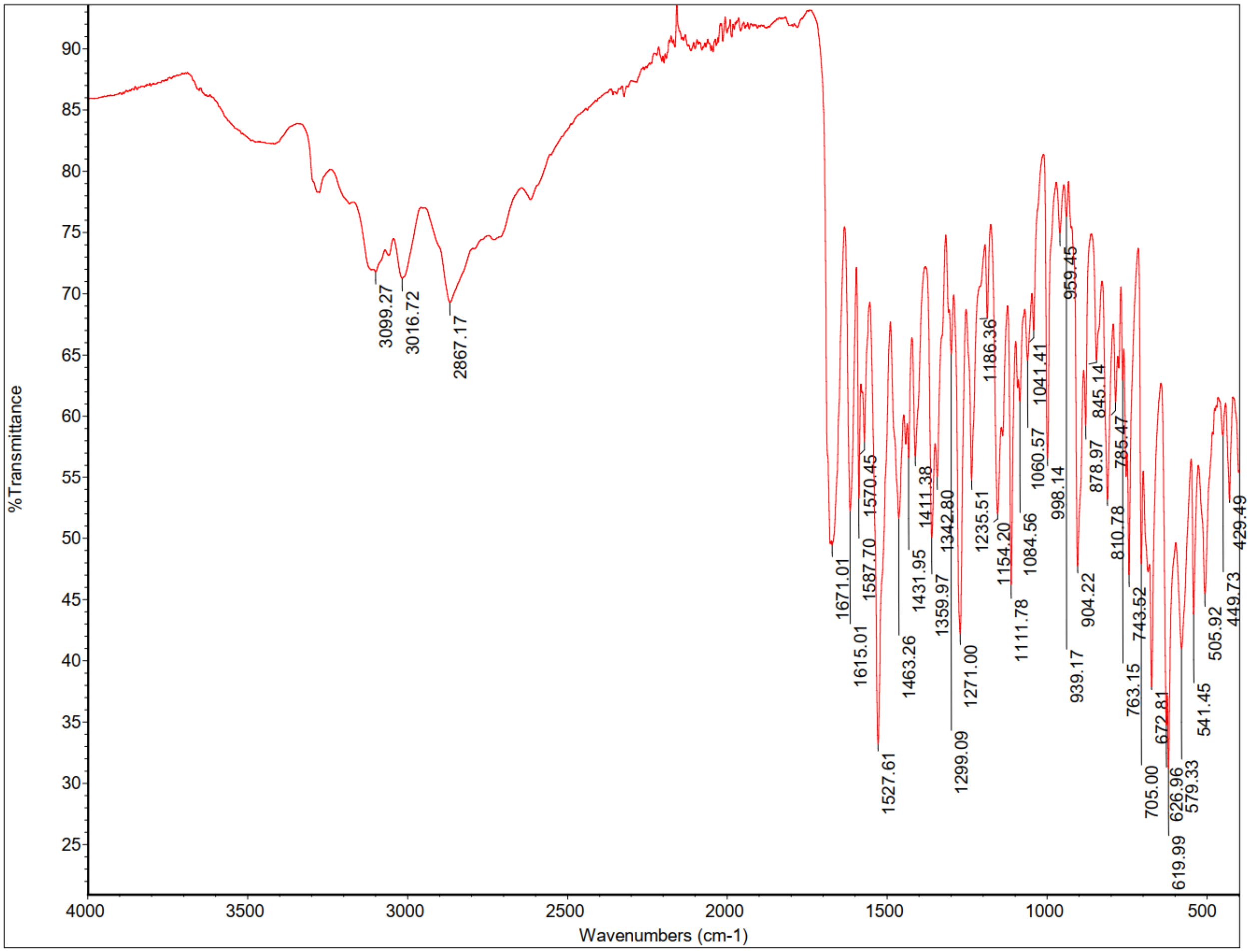

IR spectra of (*E*)-*N*'-((5-bromo-1*H*-pyrrolo[2,3-*b*]pyridin-3-yl)methylene)picolinohydrazide (**RF-87A** or **2b**) (cm^{-1}).

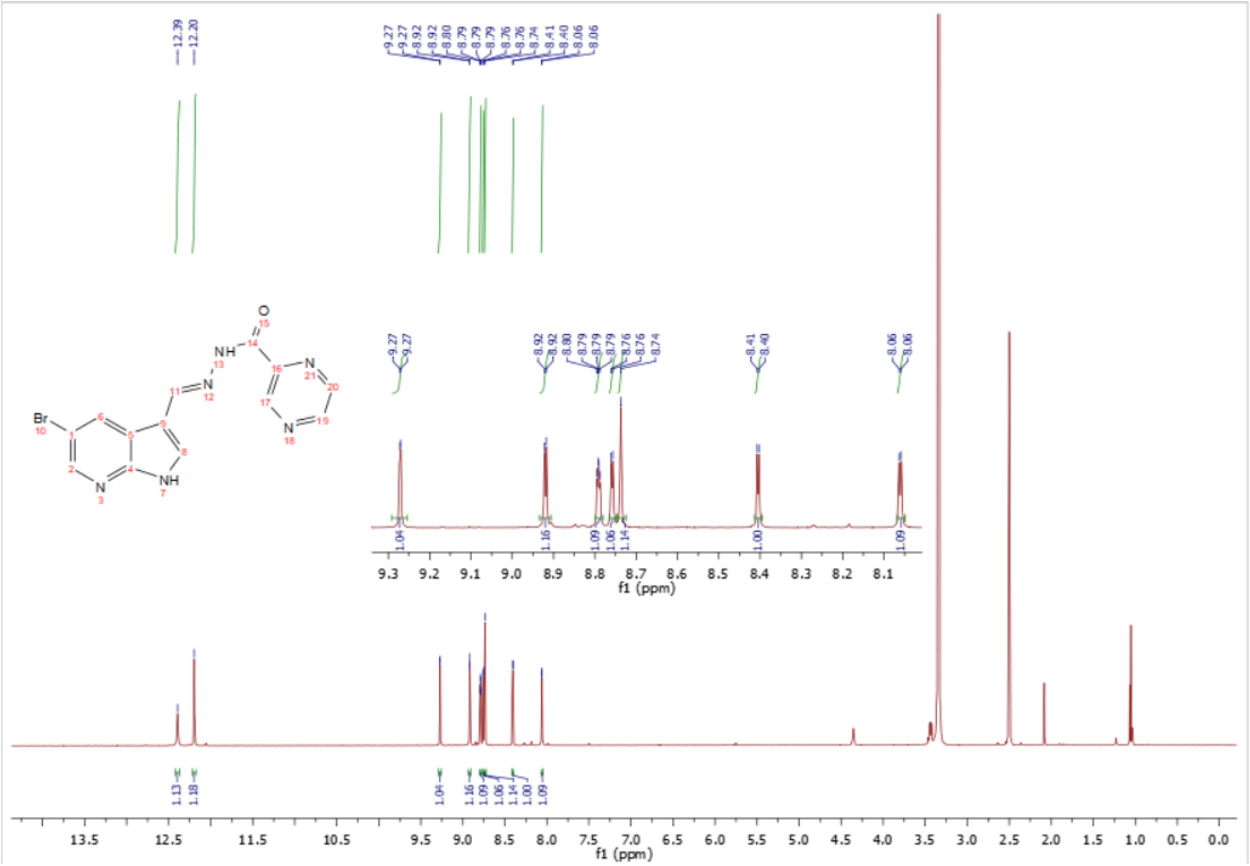

^{1}H-NMR of (*E*)-*N*'-((5-bromo-1*H*-pyrrolo[2,3-*b*]pyridin-3-yl)methylene)pyrazine-2-carbohydrazide (**RF-94A** or **2c**) (DMSO-d_6, 500 MHz).

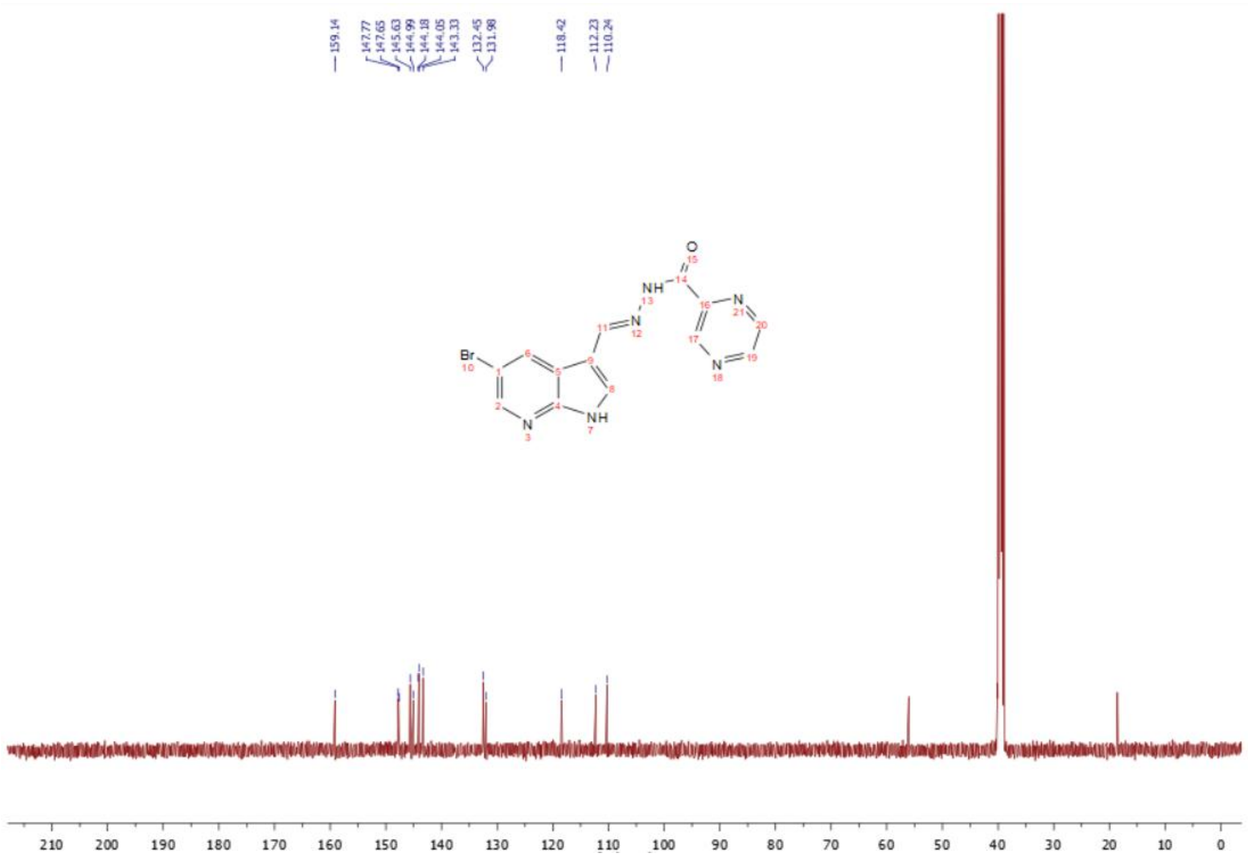

^{13}C-NMR of (*E*)-*N*'-((5-bromo-1*H*-pyrrolo[2,3-*b*]pyridin-3-yl)methylene)pyrazine-2-carbohydrazide (**RF-94A** or **2c**) (DMSO-d_6, 125 MHz).

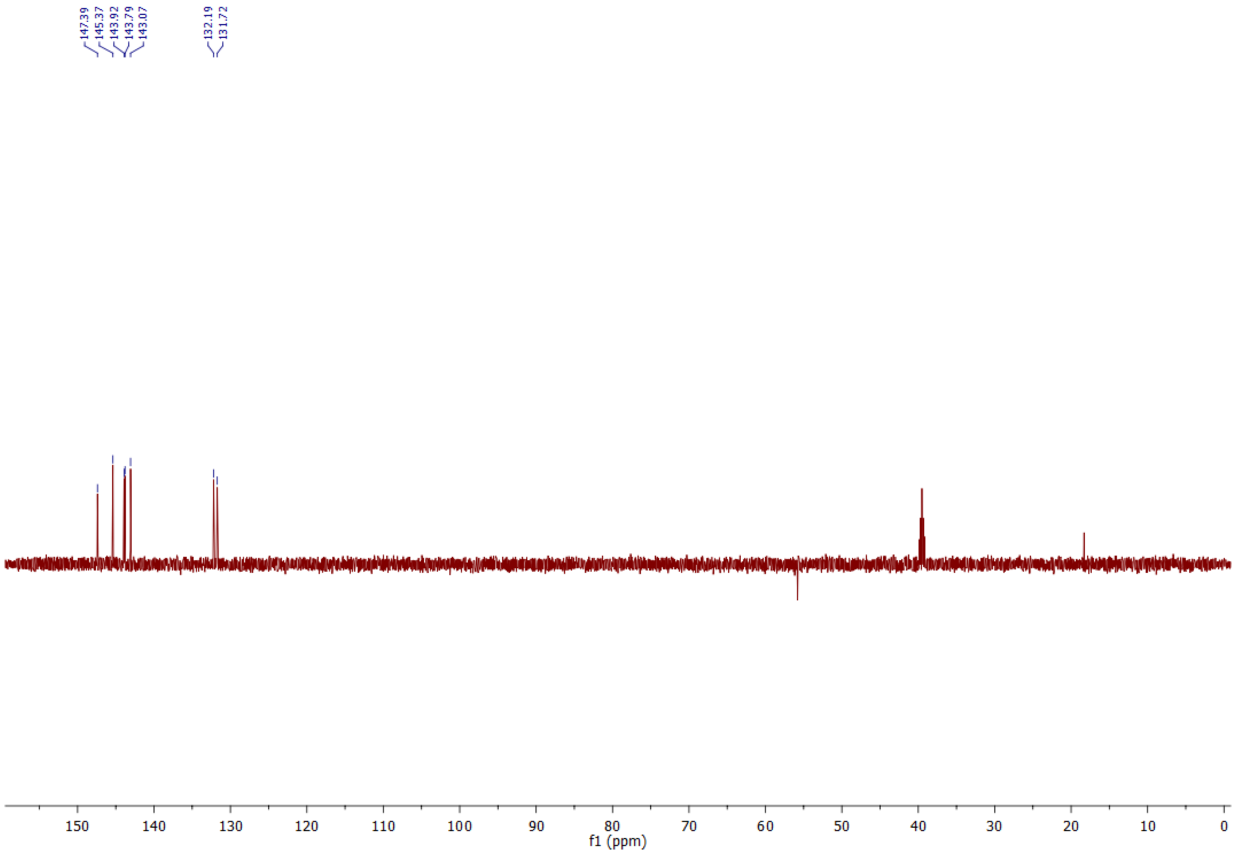

DEPT-135 experiment of (*E*)-*N*'-((5-bromo-1*H*-pyrrolo[2,3-*b*]pyridin-3-yl)methylene)pyrazine-2-carbohydrazide (**RF-94A** or **2c**) (DMSO-d_6, 60 MHz).

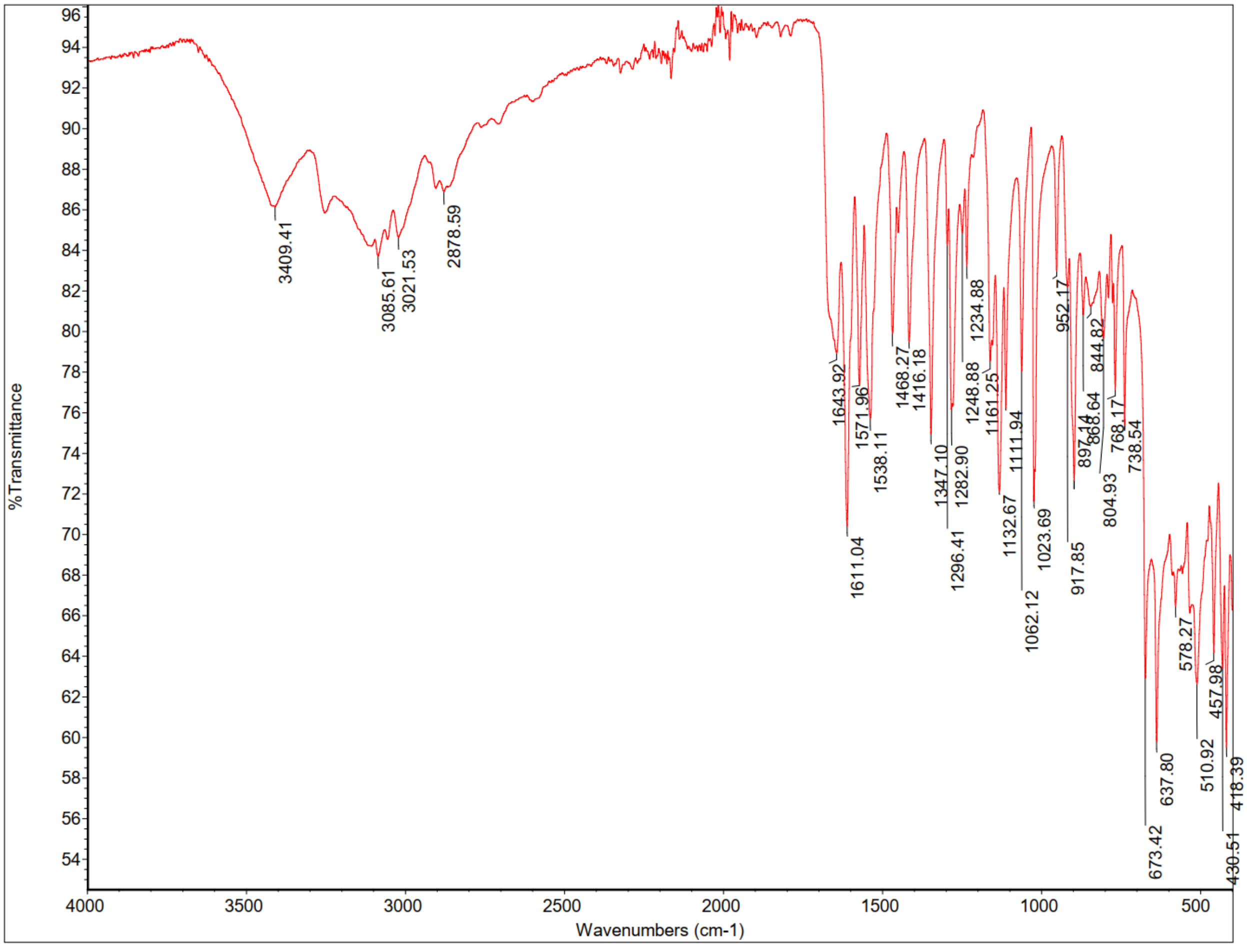

IR spectra of (*E*)-*N*'-((5-bromo-1*H*-pyrrolo[2,3-*b*]pyridin-3-yl)methylene)pyrazine-2-carbohydrazide (**RF-94A** or **2c**) (cm^{-1}).

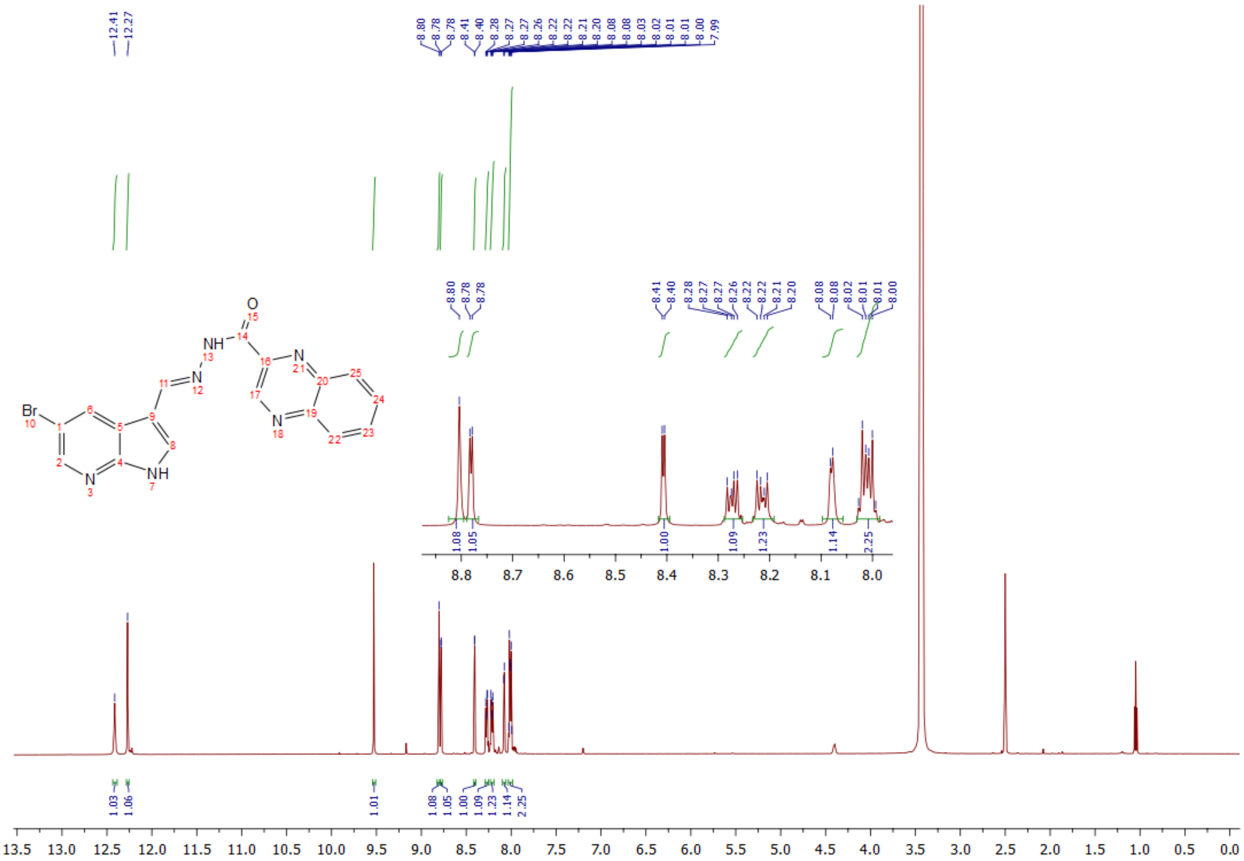

^{1}H-NMR of (*E*)-*N*'-((5-bromo-1*H*-pyrrolo[2,3-*b*]pyridin-3-yl)methylene)quinoxaline-2-carbohydrazide (**RF-94B** or **2d**) (DMSO-d_6, 500 MHz).

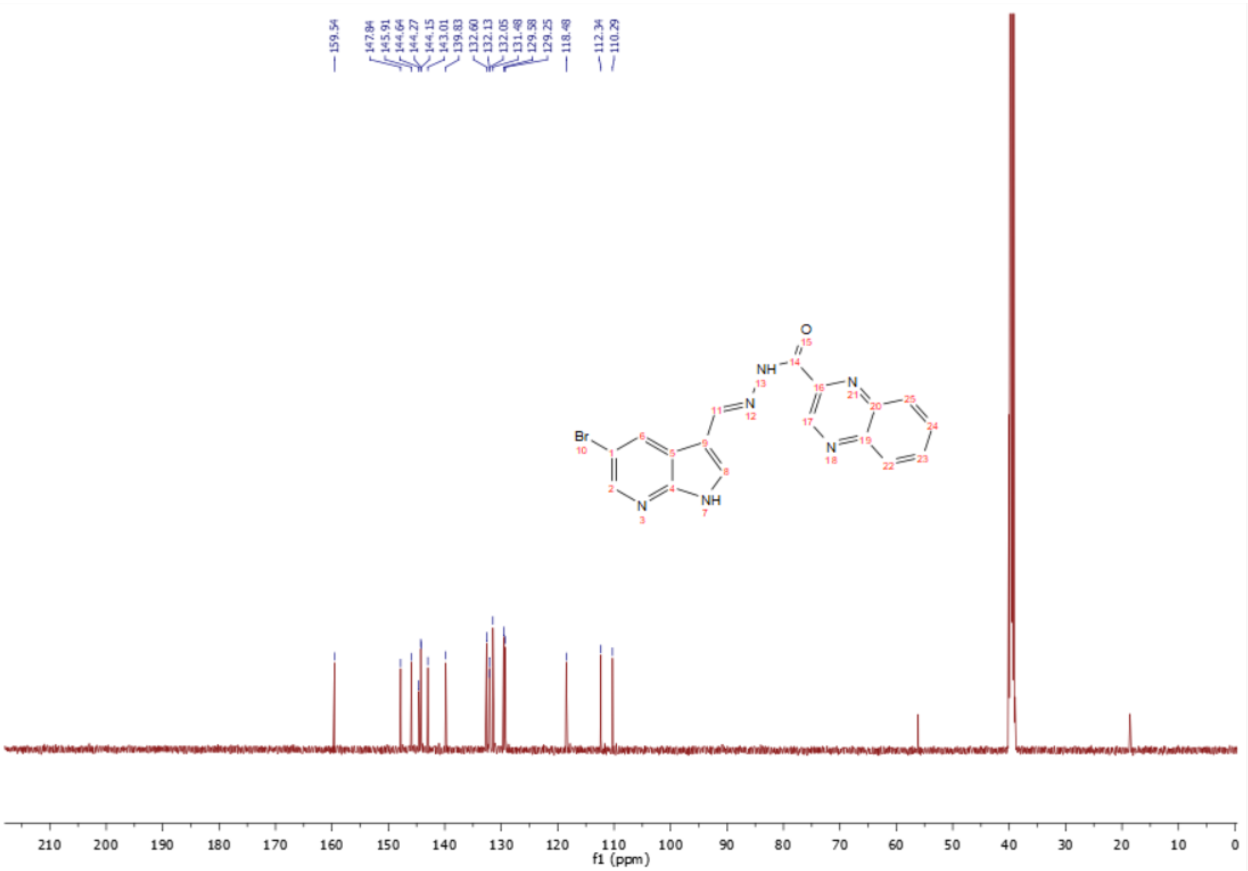

^{13}C-NMR of (*E*)-*N*'-((5-bromo-1*H*-pyrrolo[2,3-*b*]pyridin-3-yl)methylene)quinoxaline-2-carbohydrazide (**RF-94B** or **2d**) (DMSO-d_6, 125 MHz).

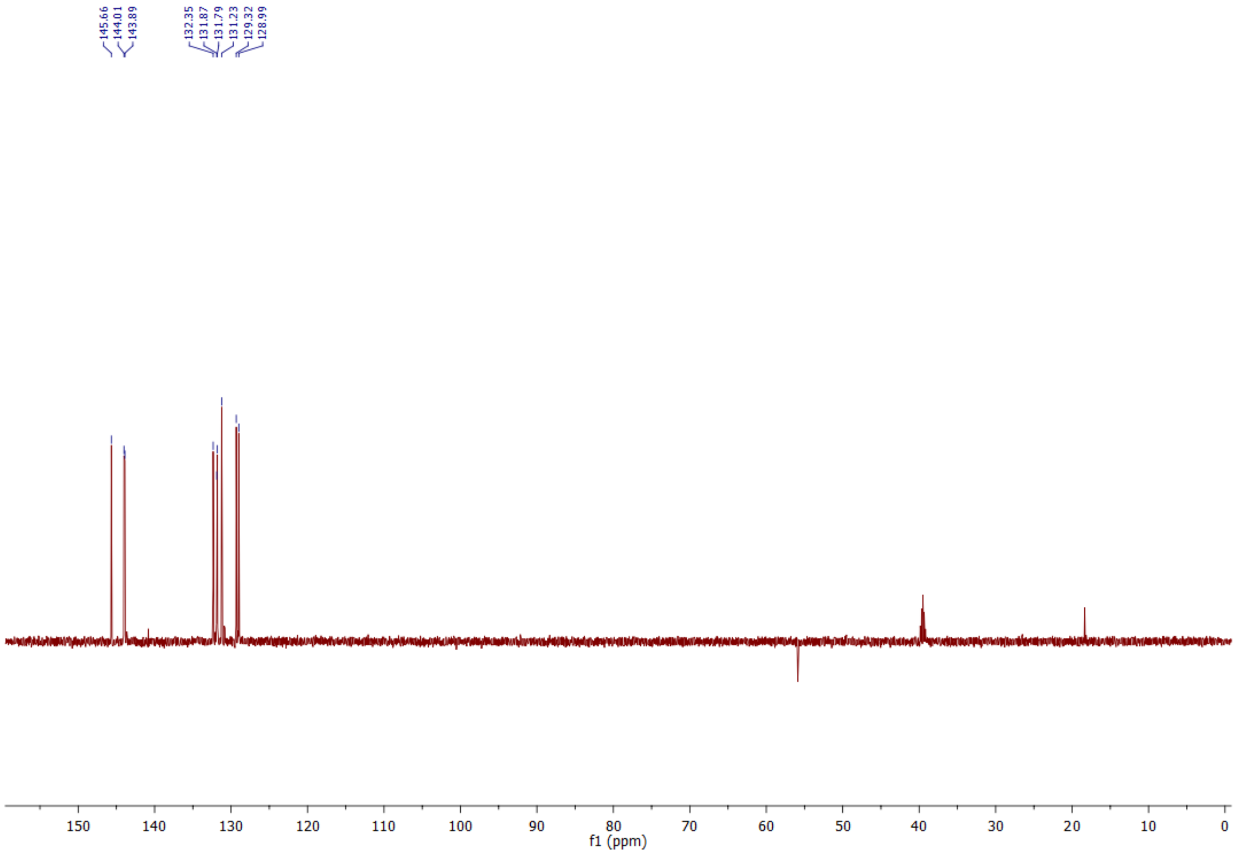

DEPT-135 experiment of (*E*)-*N*'-((5-bromo-1*H*-pyrrolo[2,3-*b*]pyridin-3-yl)methylene)quinoxaline-2-carbohydrazide (**RF-94B** or **2d**) (DMSO-d_6, 60 MHz).

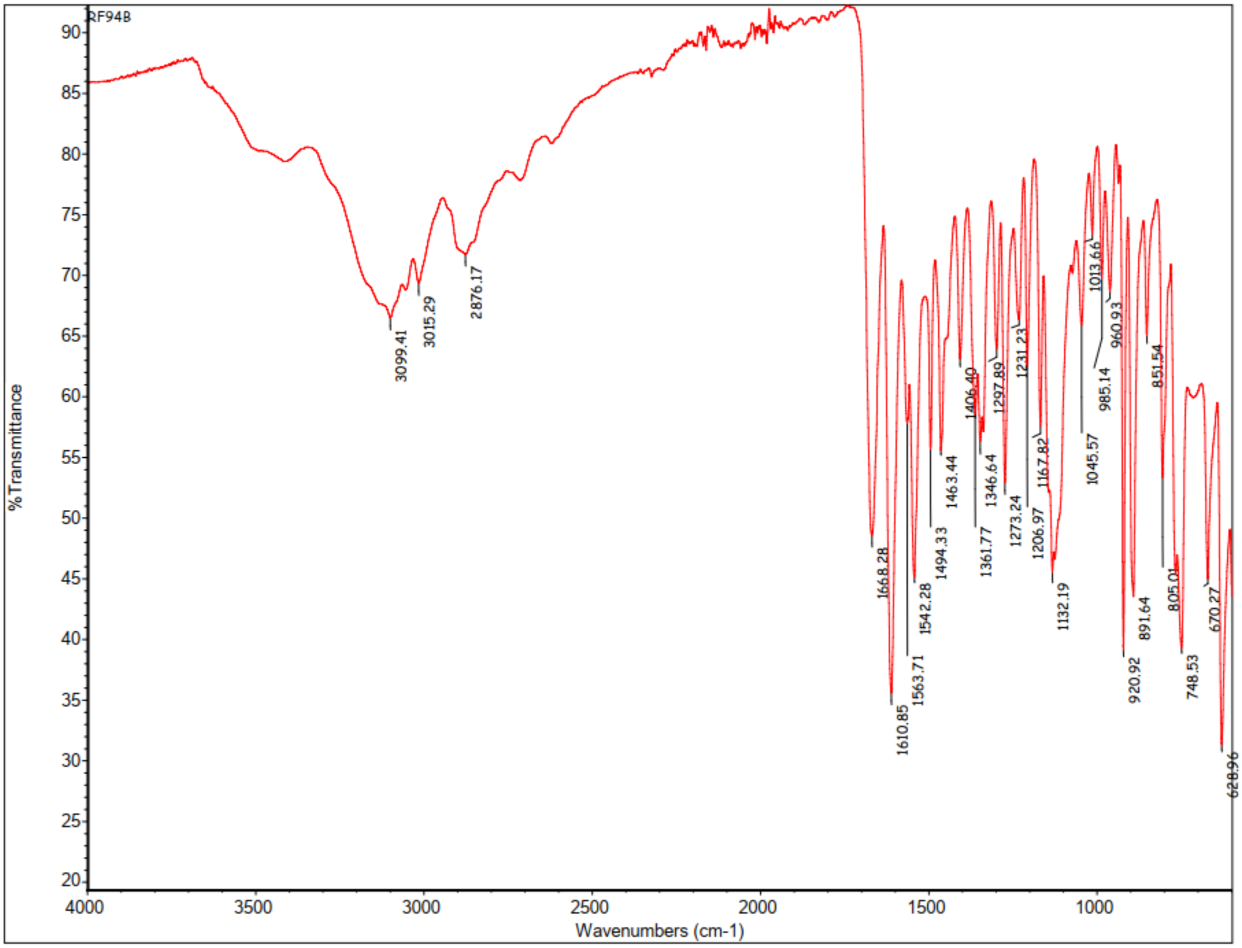

IR spectra of (*E*)-*N*'-((5-bromo-1*H*-pyrrolo[2,3-*b*]pyridin-3-yl)methylene)quinoxaline-2-carbohydrazide (**RF-94B** or **2d**) (cm^{-1}).

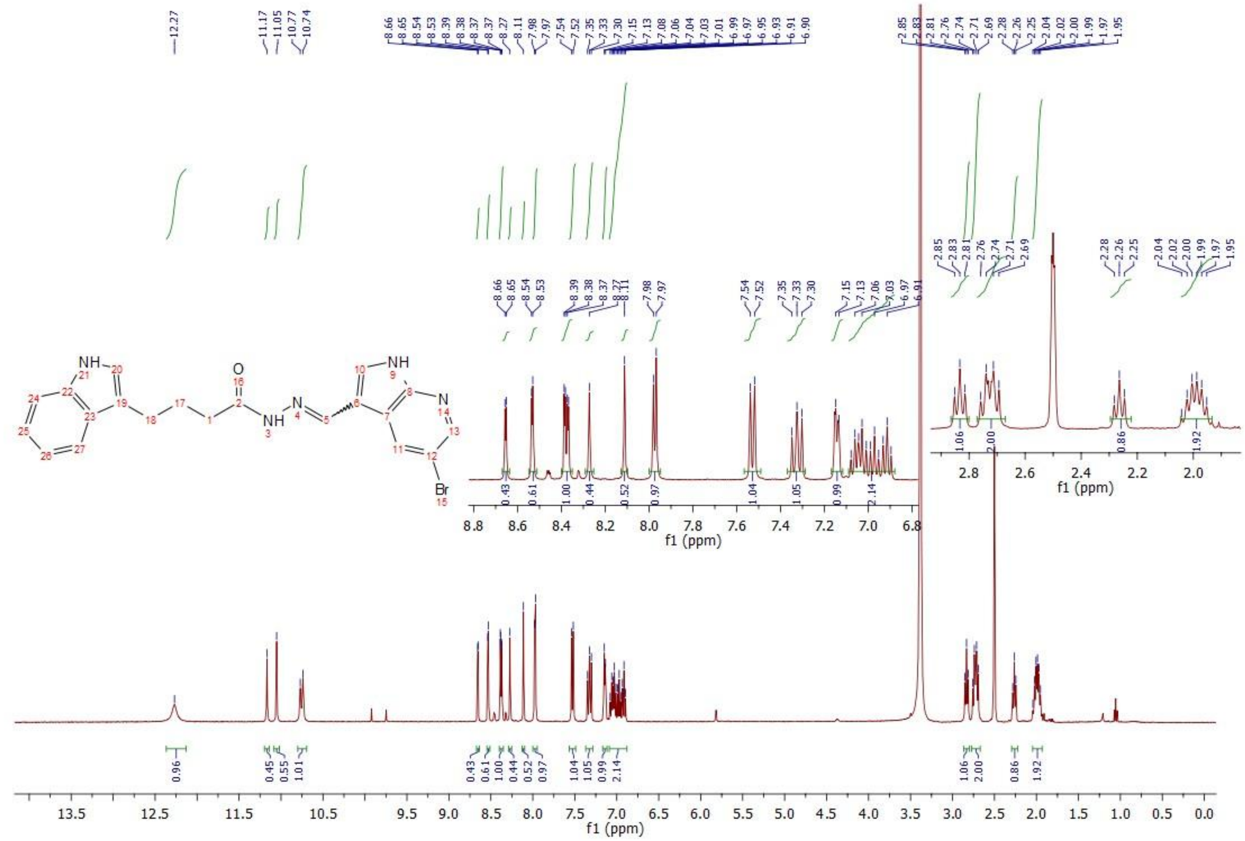

^{1}H-NMR of (*EZ*)-*N*'-((5-bromo-1*H*-pyrrolo[2,3-*b*]pyridin-3-yl)methylene)-4-(1*H*-indol-3-yl)butanehydrazide (**RF-96A** or **2e**) (DMSO-d_6, 400 MHz).

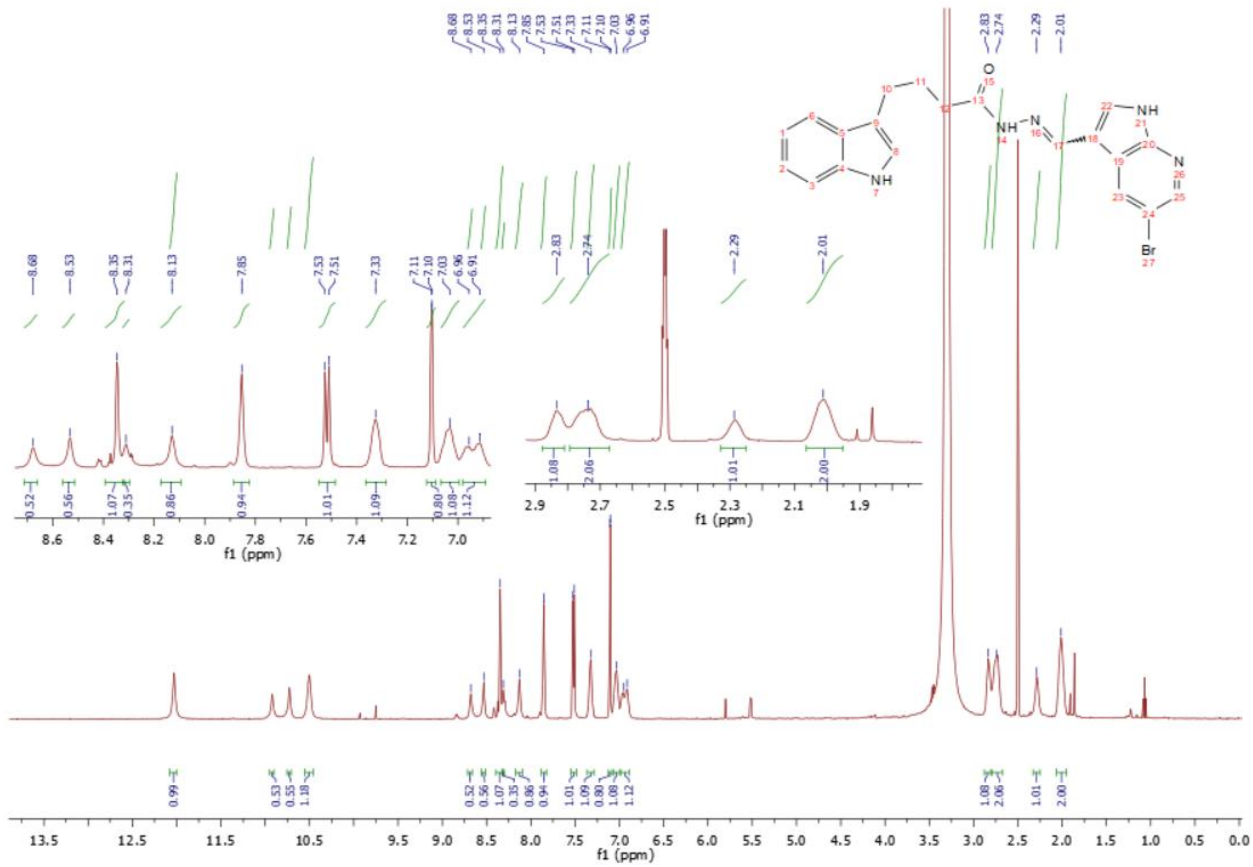

^{1}H-NMR of (*EZ*)-*N*'-((5-bromo-1*H*-pyrrolo[2,3-*b*]pyridin-3-yl)methylene)-4-(1*H*-indol-3-yl)butanehydrazide (**RF-96A** or **2e**) at 72 °C (DMSO-d_6, 500 MHz).

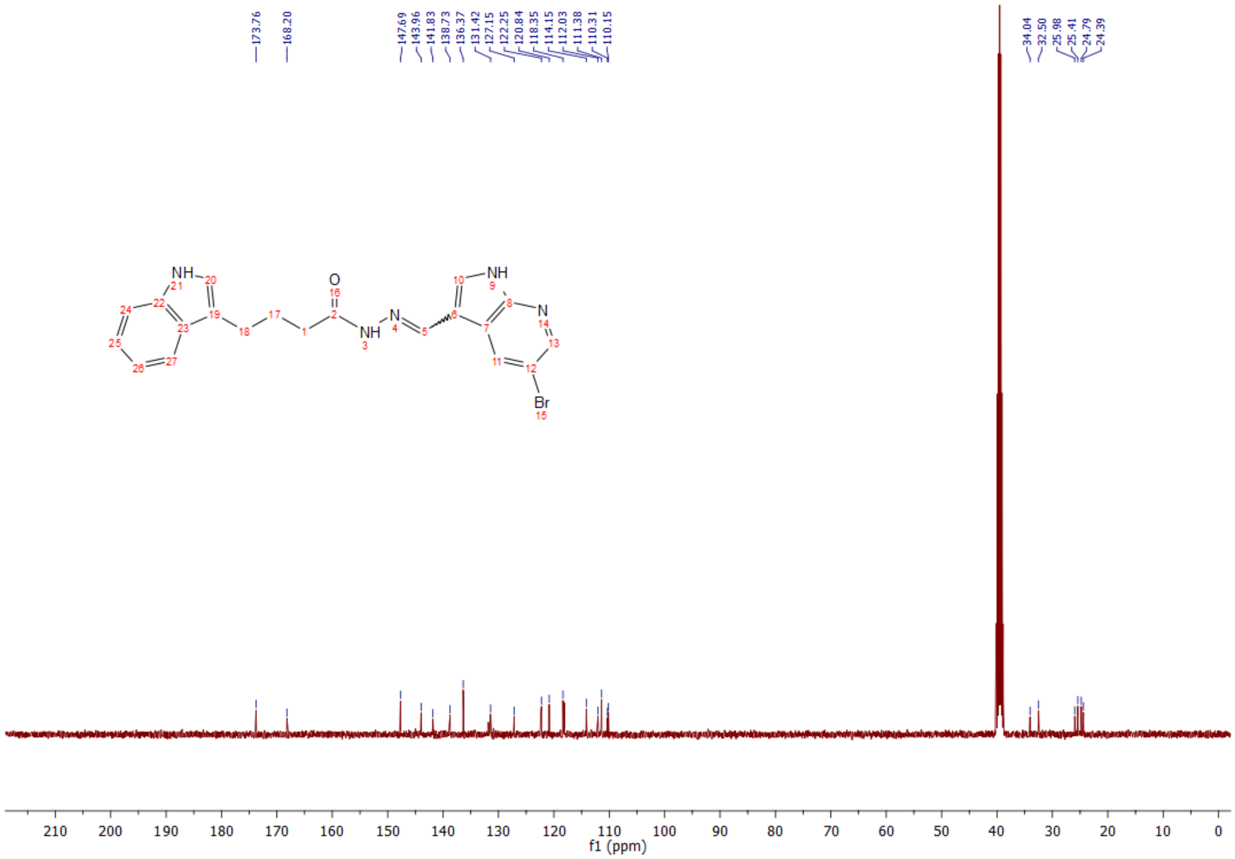

^{13}C-NMR of (*EZ*)-*N*'-((5-bromo-1*H*-pyrrolo[2,3-*b*]pyridin-3-yl)methylene)-4-(1*H*-indol-3-yl)butanehydrazide (**RF-96A** or **2e**) (DMSO-d_6, 100 MHz).

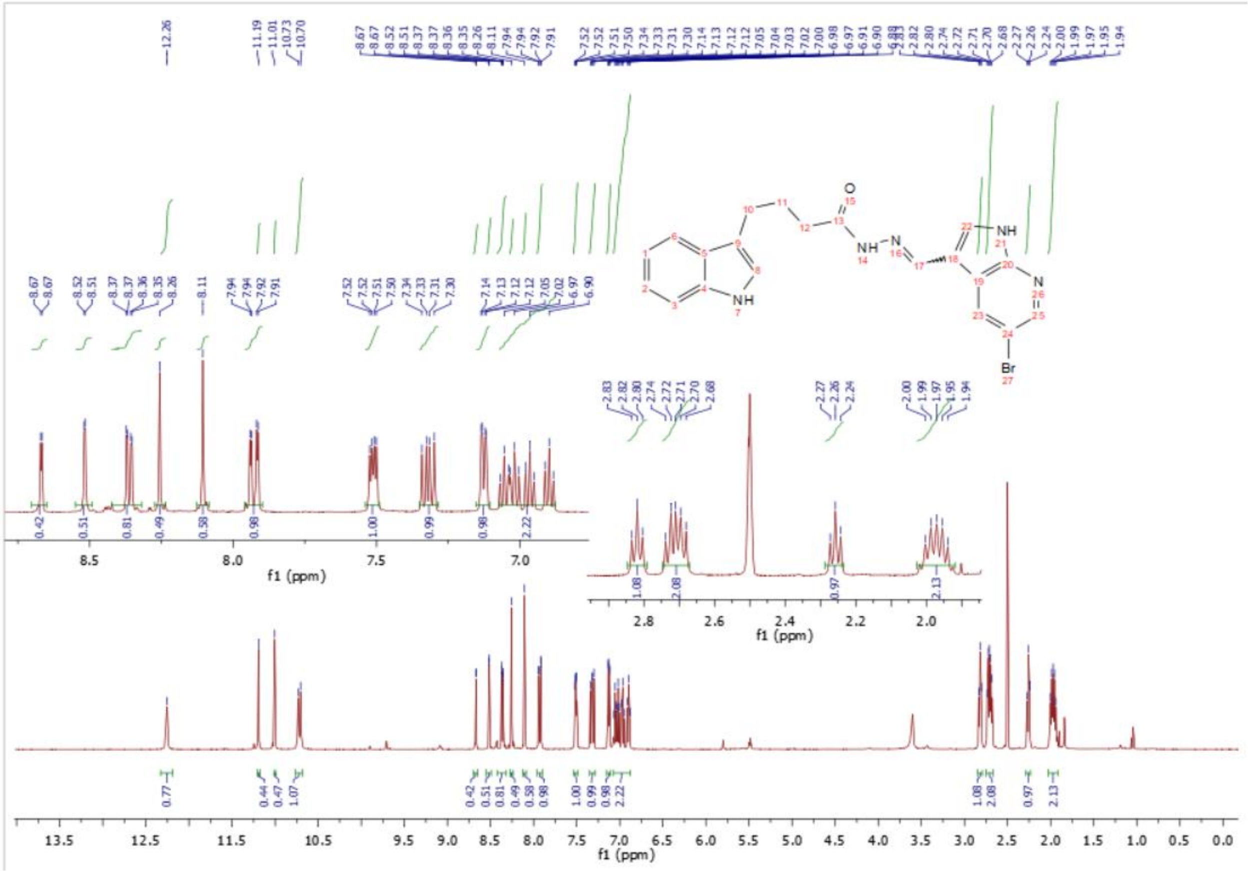

^{1}H-NMR of (*EZ*)-*N*'-((5-bromo-1*H*-pyrrolo[2,3-*b*]pyridin-3-yl)methylene)-4-(1*H*-indol-3-yl)butanehydrazide (**RF-96A** or **2e**) at 25 °C (DMSO-d_6, 500 MHz).

PRESAT

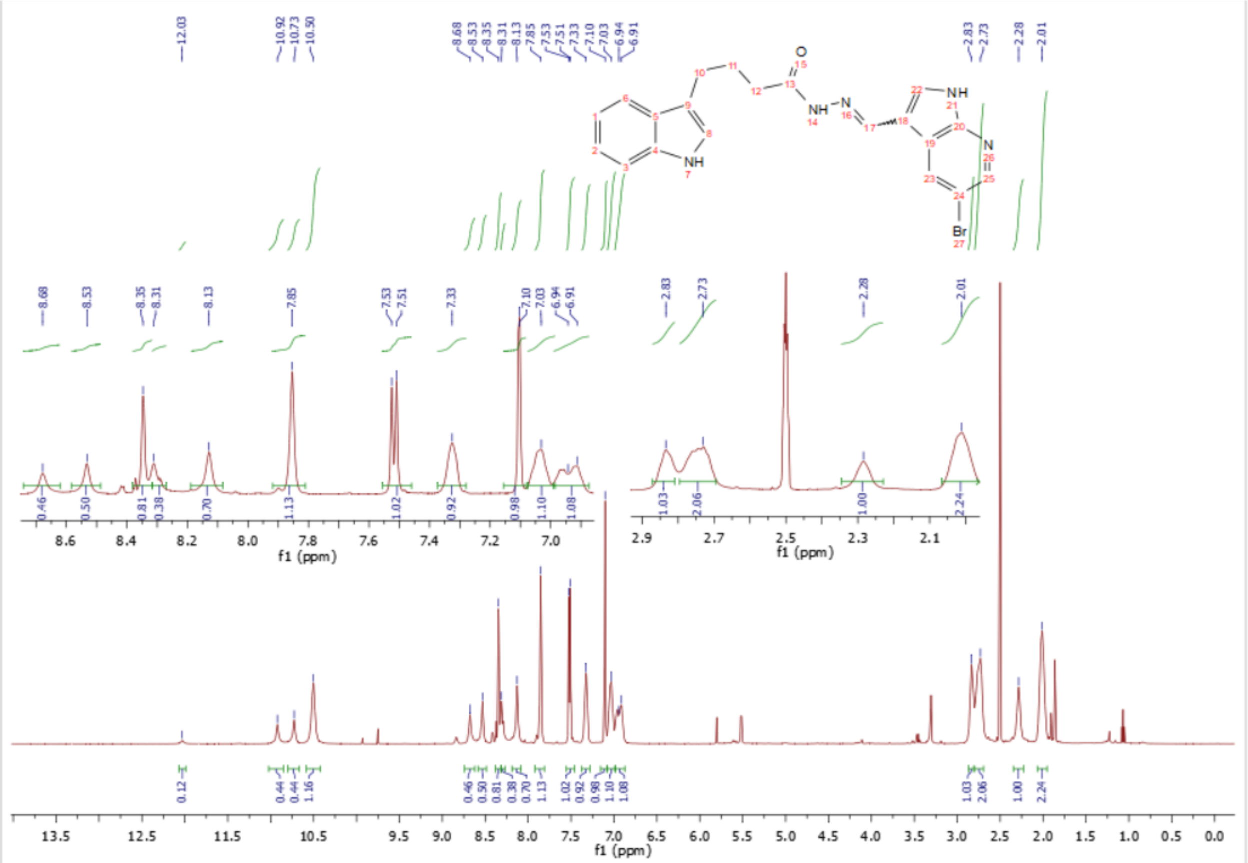

^{1}H-NMR of (*EZ*)-*N*'-((5-bromo-1*H*-pyrrolo[2,3-*b*]pyridin-3-yl)methylene)-4-(1*H*-indol-3-yl)butanehydrazide (**RF-96A** or **2e**) at 72°C (DMSO-d_6, 500 MHz).

PRESAT

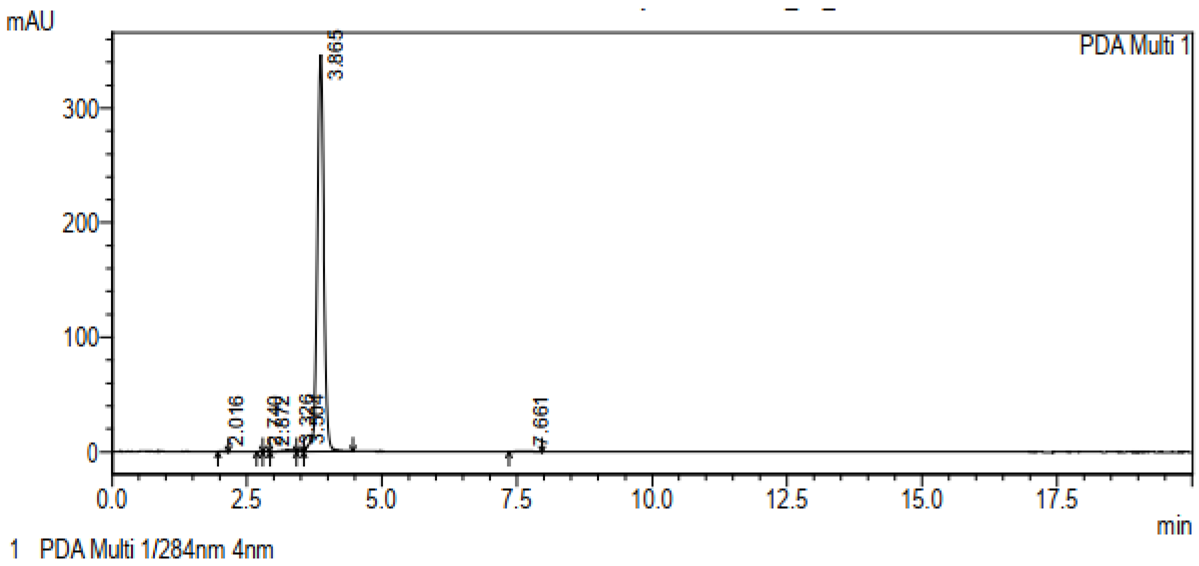

PeakTable

PDA Ch1 284nm 4nm

Peak#	Ret. Time	Area	Height	Area %	Height %
1	2.016	1016	189	0.034	0.054
2	2.740	2006	396	0.068	0.113
3	2.872	3615	682	0.122	0.194
4	3.326	29373	1709	0.994	0.486
5	3.504	13671	1842	0.463	0.523
6	3.865	2897459	346455	98.044	98.463
7	7.661	8124	590	0.275	0.168
Total		2955265	351863	100.000	100.000

Chromatogram of **RF-96A** or **2e** (batch 1)

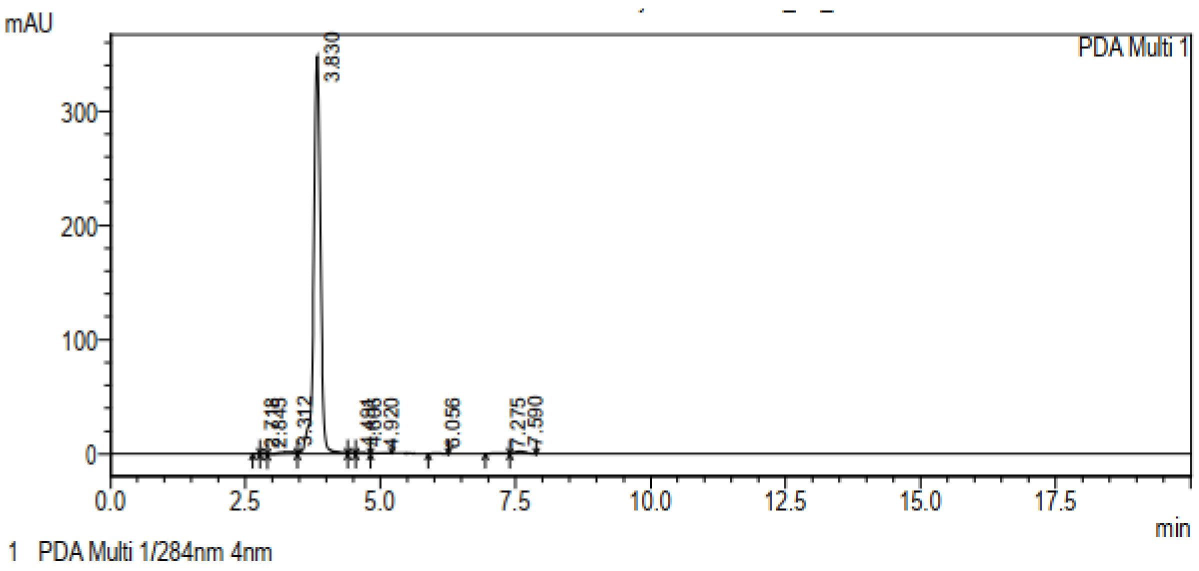

PeakTable

PDA Ch1 284nm 4nm

Peak#	Ret. Time	Area	Height	Area %	Height %
1	2.718	1834	336	0.059	0.095
2	2.845	2820	540	0.090	0.152
3	3.312	38223	1755	1.223	0.494
4	3.830	3017954	347596	96.588	97.812
5	4.491	8635	997	0.276	0.281
6	4.606	12758	1036	0.408	0.292
7	4.920	6354	563	0.203	0.158
8	6.056	1387	127	0.044	0.036
9	7.275	8444	500	0.270	0.141
10	7.590	26149	1920	0.837	0.540
Total		3124557	355371	100.000	100.000

Chromatogram of **RF-96A** or **2e** (batch 2)

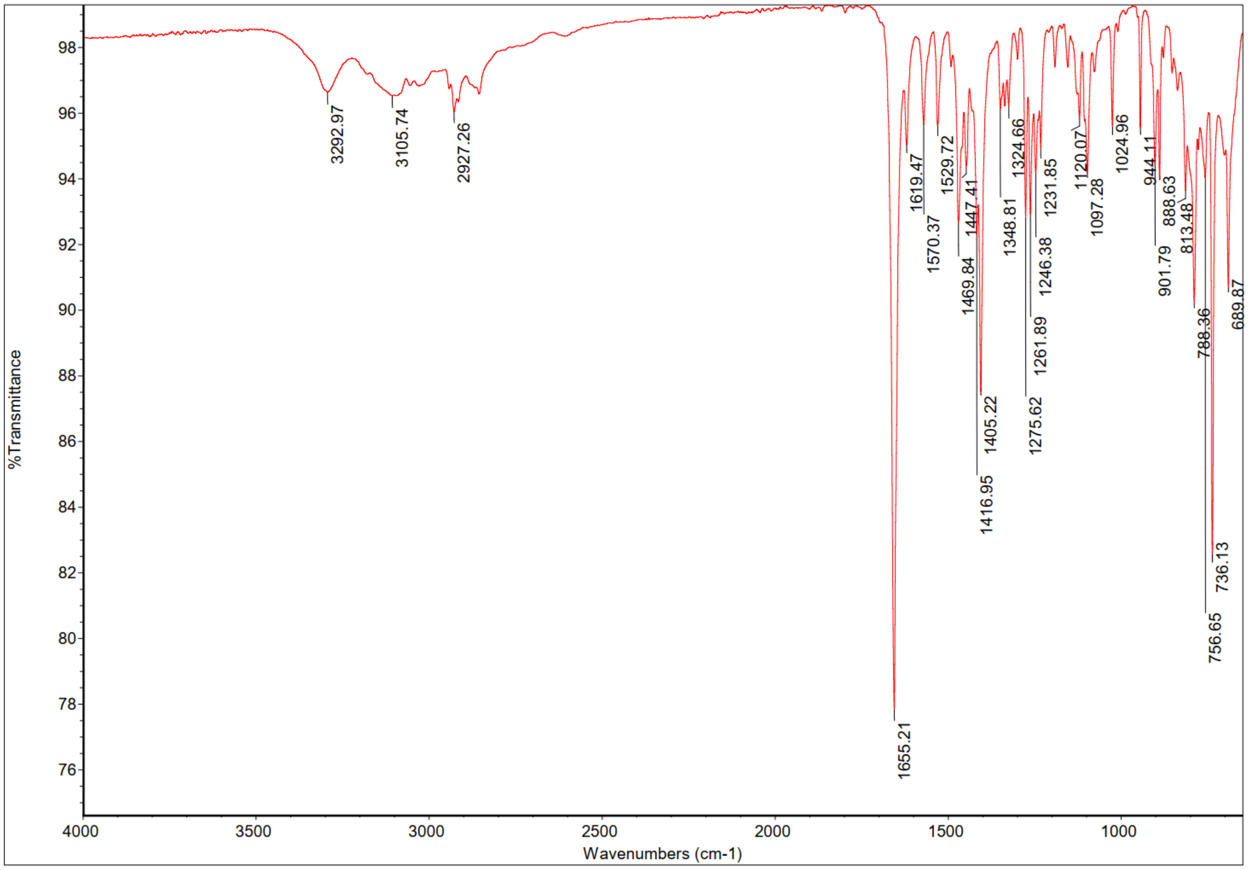

IR spectra of (*EZ*)-*N*'-((5-bromo-1*H*-pyrrolo[2,3-*b*]pyridin-3-yl)methylene)-4-(1*H*-indol-3-yl)butanehydrazide (**RF-96A** or **2e**) (cm^{-1}).
